# Unraveling Energy Flow Mechanisms in Semiconductors by Ultrafast Spectroscopy: Germanium as a Case Study

**DOI:** 10.1002/advs.202515470

**Published:** 2026-01-12

**Authors:** Grazia Raciti, Begoña Abad, Riccardo Dettori, Raja Sen, Aswathi K. Sivan, Jose M. Sojo‐Gordillo, Nathalie Vast, Riccardo Rurali, Claudio Melis, Jelena Sjakste, Ilaria Zardo

**Affiliations:** ^1^ Department of Physics University of Basel Basel 4056 Switzerland; ^2^ Department of Physics University of Cagliari Monserrato CA 09042 Italy; ^3^ SATIE, CNRS, ENS Paris‐Saclay Université Paris‐Saclay Gif‐sur‐Yvette 91190 France; ^4^ Laboratoire des Solides Irradiés, CEA/DRF/IRAMIS, Ecole Polytechnique, CNRS Institut Polytechnique de Paris Palaiseau 91128 France; ^5^ Institut de Ciència de Materials de Barcelona ICMAB–CSIC Campus UAB Bellaterra 08193 Spain; ^6^ Swiss Nanoscience Institute University of Basel Basel 4056 Switzerland

**Keywords:** coherent phonons, electron–phonon dynamics, germanium, phonon temperature, ultrafast spectroscopy

## Abstract

Semiconductor materials are the foundation of modern electronics, and their functionality is dictated by the interactions between fundamental excitations occurring on (sub‐)picosecond timescales. Using time‐resolved Raman spectroscopy and transient reflectivity measurements, the ultrafast dynamics in germanium are elucidated. An increase in the optical phonon temperature is observed in the first few picoseconds, driven by the energy transfer from photoexcited holes, and the subsequent decay into acoustic phonons through anharmonic coupling. Moreover, the temperature, Raman frequency, and linewidth of this phonon mode show strikingly different decay dynamics. This difference is ascribed to the local thermal strain generated by the ultrafast excitation. Brillouin oscillations are also observed, given by a strain pulse traveling through germanium, whose damping is correlated to the optical phonon mode. These findings, supported by density functional theory and molecular dynamics simulations, provide a better understanding of the energy dissipation mechanisms in semiconductors.

## Graphical abstract

A multi‐technique pump‐probe setup capable of performing both transient reflectivity and time‐resolved Raman spectroscopy after ultrafast excitation, provides a comprehensive understanding of ultrafast electronic and phononic processes. Distinct decay rates for the temperature, frequency, and linewidth of the phonon mode in germanium are observed, along with the propagation of coherent acoustic phonon oscillations.

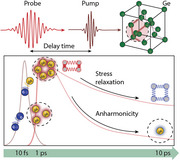

## 1 Introduction

Semiconductor devices are the building blocks of most modern technologies. Their speed, efficiency, and performance are directly related to the energy transfer processes during external excitations. More specifically, the charge and heat carrier dynamics and their mutual interaction are the fundamental mechanisms that govern their macroscopic physical properties and, consequently, set limits to their operation conditions. Therefore, a fundamental understanding of the energy decay channels after excitation enables improved device performance,^[^ ^1^ ^]^ advancing heat management of next‐generation devices such as solar cells and optical detectors. In particular, germanium (Ge) is a key material for the semiconductor industry, with applications spanning a wide range, from electronics^[^ ^2^, ^3^ ^]^ to photonics^[^ ^4^ ^]^ to quantum technologies.^[^ ^5^ ^]^ Ge is a group IV semiconductor with an indirect bandgap, which attracts much attention due to its high carrier mobility^[^ ^6^ ^]^ and compatibility with silicon‐based systems. Although this material has been extensively researched and many of its properties have been well characterized for decades, the fundamental mechanisms governing energy dissipation in Ge are still poorly understood. Indeed, to date, only a handful of studies exploring the carrier and phonon dynamics after ultrafast excitation in Ge are available^[^ ^7^, ^8^, ^9^, ^10^ ^]^ and cannot fully describe the mechanisms behind it. Technological and computational progresses enable now to probe dynamics with an unprecedented time and energy resolution.

In particular, carrier and phonon dynamics in Ge have been studied by time‐resolved Raman spectroscopy (TRRS), a pump‐probe spectroscopy technique that probes incoherent phonon lifetimes.^[^ ^11^ ^]^ These studies are mainly divided into low and high photoexcited carrier densities n, namely n <10^20^ cm^−3^ and n >10^20^ cm^−3^, respectively. In general, after ultrafast pump excitation, electrons (holes) will initially be excited to high‐energy states on a timescale of femtoseconds. These carriers redistribute energy among themselves via carrier‐carrier scattering in tens of femtoseconds, forming a hot Fermi‐Dirac distribution. These hot electrons (holes), which have a mean kinetic energy considerably higher than the thermal energy of the lattice, will relax to the bottom (top) of the conduction (valence) band by emitting optical and acoustic phonons in a few picoseconds. Subsequently, they reach thermal equilibrium with the lattice in about 2–100 ps, after which carrier recombination occurs.^[^ ^12^, ^13^ ^]^

At moderate photoexcited hole densities, Young et al. studied the lifetime of the non‐equilibrium longitudinal (LO) and transversal (TO) optical phonons in Ge. Both LO and TO phonons were shown to have a lifetime of 4 ps at room temperature. The fact that both phonon populations were similar was ascribed to the generation of phonons by heavy holes in the vicinity of the zone center.^[^ ^14^ ^]^ A year later, Othonos et al. used a combination of TRRS and transient reflectivity (TR) to study the hot‐carrier dynamics in Ge.^[^ ^15^ ^]^ Both works found that non‐equilibrium optical phonon populations in Ge are effectively generated and probed by TRRS. At high photoexcited hole densities, Ledgerwood et al. found that phonon reabsorption by holes is responsible for faster optical phonon decay times (<3 ps) than previously demonstrated for lower photoexcited densities.^[^ ^16^, ^17^ ^]^ Several studies on various semiconductors have also determined this decay, showing values of 7 ps (GaAs),^[^ ^13^ ^]^ 1.7 ps (Si),^[^ ^18^ ^]^ 2.2 ps (graphite),^[^ ^19^ ^]^ 0.35 ps (GaN),^[^ ^20^ ^]^ for relatively high photoexcited carrier densities (n >10^19^ cm^−3^). While the absolute value of these time constants is material‐dependent, the anharmonic coupling in between optical and acoustic phonons is a general phenomenon occurring in all semiconductors after ultrafast excitation, which is critical to understand their energy dissipation mechanisms.

However, the time resolution of the studies performed in Ge was limited by the pulse duration of 4 ps, so only phonon lifetimes longer than this pulse duration could be probed. Shorter lifetimes at higher photoexcited carrier densities were extracted from the linewidth broadening observed during time‐averaged anti‐Stokes measurements. In this technique, Raman spectra are probed with a picosecond probe pulse without pump excitation.^[^ ^17^ ^]^ The lifetime extracted from this broadening may not be strictly comparable to the lifetime measured by time‐resolved measurements, as time‐averaging does not follow the phonon population after a single excitation. Therefore, more precise time‐resolved Raman spectroscopy measurements with pump and probe pulse durations shorter than the expected phonon lifetime are essential for understanding phonon dynamics.

Raman spectroscopy also enables the study of the phonon self‐energy, i.e., phonon frequency and lineshape broadening. In group IV semiconductors, such as Ge, the interaction between carriers and phonons takes place through the deformation potential.^[^ ^21^ ^]^ This mechanism may lead to a contribution of the photoexcited carriers to the phonon self‐energy.^[^ ^17^ ^]^ In particular, at high photoexcited hole densities, single‐particle excitations may play a role in the renormalization of the phonon energy, similarly to the line broadening and frequency shift in highly p‐doped Ge as compared to intrinsic Ge.^[^ ^22^ ^]^ On the other hand, pump‐induced strain may also affect the phonon self‐energy.^[^ ^23^ ^]^ Extraordinary advances in ultrafast laser and detection technology, as well as progress in computational techniques allow now to experimentally address the dynamics of the phonon self‐energy and phonon temperature in Ge with improved accuracy and temporal resolution. Therefore, a systematic study of the ultrafast dynamics of energy dissipation channels in Ge is needed to shed light on the fundamental mechanisms that will enable improved devices. Understanding how these processes evolve is critical for the optimal design of Ge‐based semiconductor nanocomponents.

In this work, we employ TRRS and TR to study the energy dissipation dynamics following ultrafast laser excitation on a (111)‐oriented bulk Ge sample. In particular, we study the optical phonon population and its temperature dynamics within the first few picoseconds after ultrafast excitation, observing both the rise and its subsequent decay. We extract the relaxation decay time for all Raman spectral features – i.e., intensity, Raman frequency, and linewidth – and compare our experimental results with molecular dynamics (MD) and density functional theory (DFT), finding excellent agreement. Finally, we probe the correlation between optical phonon modes and coherent acoustic waves excited in the system.

## 2 Results

### 2.1 Phonon Dynamics in Germanium

**Figure 1:**
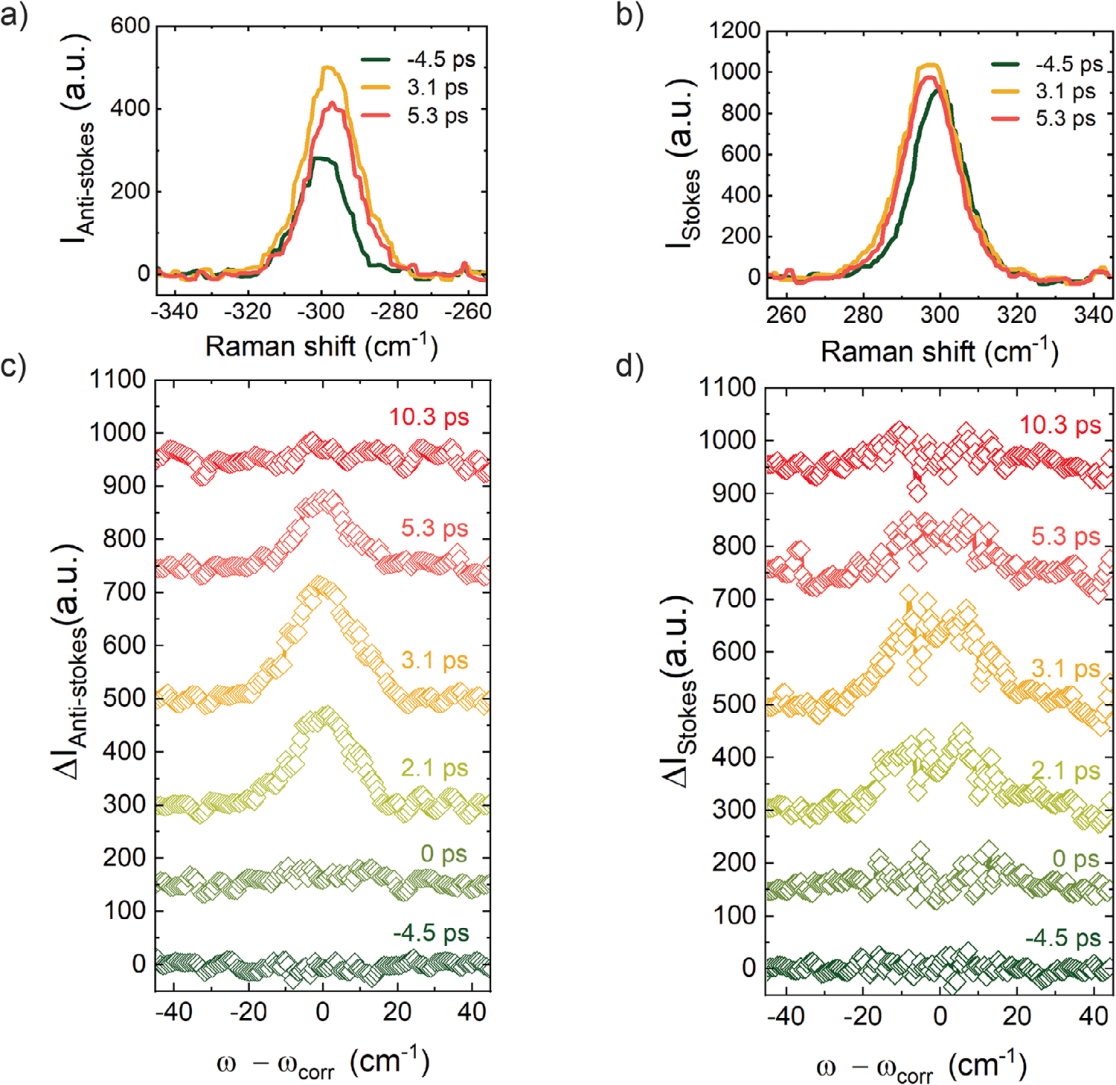
Time‐resolved spontaneous Raman spectroscopy. Raman signal of the germanium TO/LO phonon mode detected at selected delay times before and after pump excitation at a carrier density of 1.5 · 10^20^cm^−3^. Upper panels: Raman spectra for three representative times showing both the change in intensity and phonon frequency for the anti‐Stokes (a) and Stokes (b) bands. Negative (positive) times correspond to spectra measurements before (after) the arrival of the pump pulse. Lower panels: difference Raman spectra for anti‐Stokes (c) and Stokes (d) scattering, extracted by subtracting the average spectra taken at negative delay times. To account for spectral drift as a function of time, prior to the subtraction, each spectrum is offset by Δω_*corr*_, which is the difference between its peak position and that of the average Raman shift of all spectra before excitation.

We perform TRRS (described in Experimental Section) on an intrinsic crystalline Ge sample oriented along the 〈111〉 direction. In brief, an ultrafast pump laser pulse brings the system out of equilibrium, and a subsequent probe pulse monitors the evolution of the Raman spectrum as a function of delay time. Therefore, we track the dynamics of the TO/LO phonon mode of Ge (see Experimental Section and Section S1, Supporting Information, for further details of the experimental setup, and experimental conditions). As Raman spectroscopy probes phonon modes near the Γ‐point, TRRS is particularly well suited to explore phonon dynamics in Ge, since the energy deposited in carriers after ultrafast excitation flows preferentially to low wavevectors due to the conservation of energy and momentum.^[^ ^7^ ^]^ **Figure** **1**a,b show the TO/LO mode intensity, located at ± 300.7 cm^−1^,^[^ ^24^ ^]^ of the anti‐Stokes (AS), and Stokes (S) Raman scattering, respectively, at various representative times for an excited carrier density of 1.5 · 10^20^ cm^−3^. Although both bands undergo an increase in intensity with time, the AS scattering shows a larger change in relative intensity (Δ*I*/*I*) than that of the S scattering, gaining up to 45% of intensity at 3.5 ps. Instead, the maximum change in intensity for the S band is only 13% at the same delay time. This is expected from the dependence of the S and AS scattering on the phonon population, which is *n* + 1 and *n*, respectively.^[^ ^11^ ^]^ Although the AS scattering is less likely to occur at equilibrium, i.e., near room temperature, because it requires pre‐existing excited phonons, the ultrafast excitation increases the population of high‐energy phonons, causing a larger relative increase in the AS intensity as compared to the S intensity. Moreover, Figure 1a,b also show a clear red shift for both bands as a function of time.

To better visualize the change in intensity as a function of time, Figure 1c and d show the difference Raman spectra at various delay times before and after pump excitation for both AS and S, respectively (see Section S1.2, Supporting Information, for further details on this subtraction). Despite Δ*I*/*I* being very different for both bands, their dynamics are very similar: there is an initial increase in intensity in the vicinity of the optical phonon response located at ± 301cm^−1^, which reaches a maximum at 3.5 ps and eventually decreases until it fully vanishes for *t* >10 ps.

**Figure 2:**
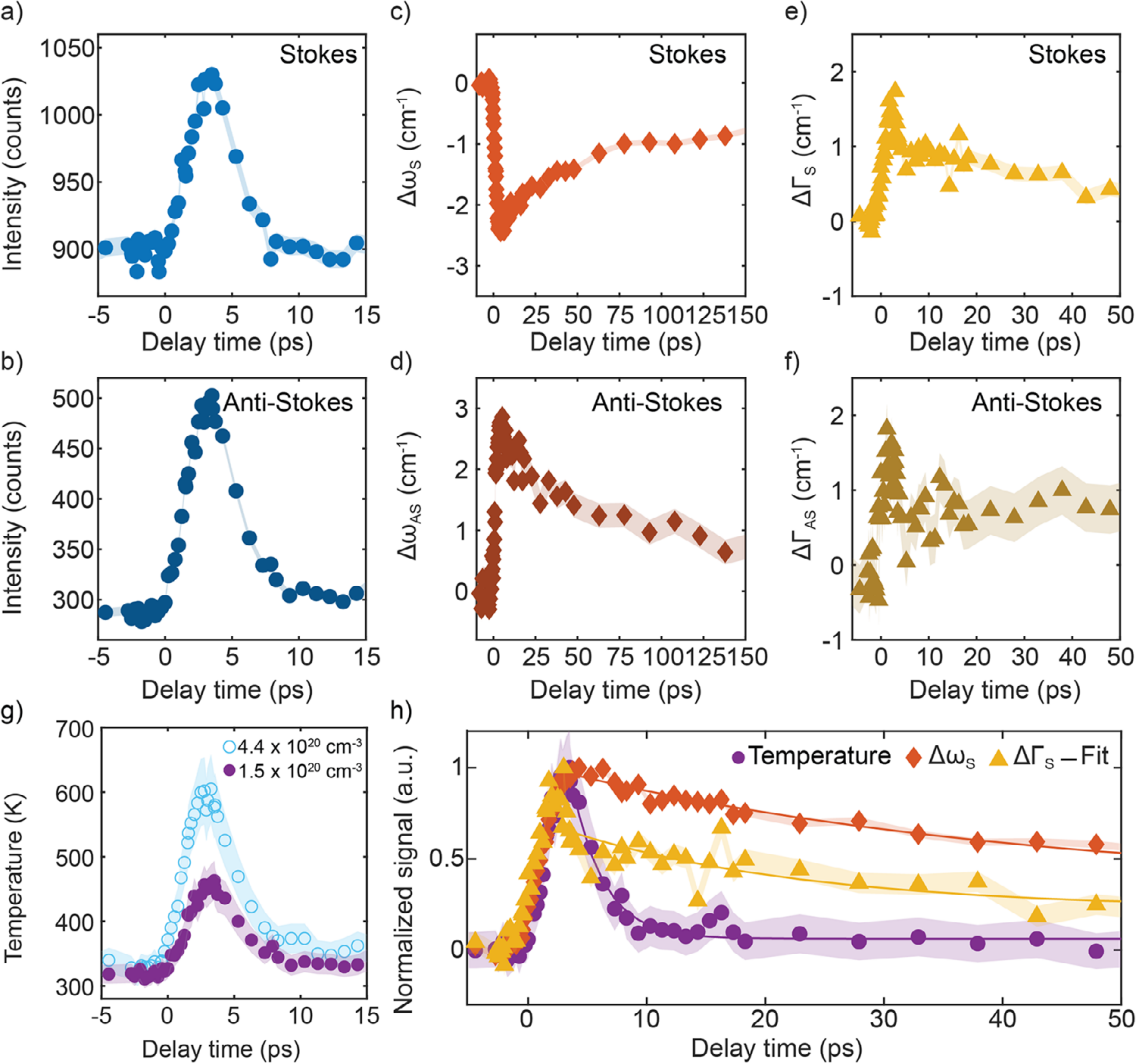
Dynamics of the Ge TO/LO phonon mode. Spectral features are extracted by fitting the Raman peak to a Gaussian fit at the acquired delay times. a,b) Intensity extracted from the Gaussian fit of Stokes (a) and Anti‐Stokes (b). c,d)  Change in Raman frequency shift (Δω) of Stokes (c) and Anti‐Stokes (d). e,f) Change in linewidth (ΔΓ) extracted for Stokes (e) and Anti‐Stokes (f) scattering, respectively. g) Temperature of the phonon mode calculated from the anti‐Stokes/Stokes intensity ratio for low and high excitation carrier densities: 1.5 · 10^20^ and 4.4 · 10^20^cm^−3^, respectively. There is a rise corresponding with the building up of the TO/LO mode, followed by a decay with a time constant of 2.7 ± 0.2 and 2.3 ± 0.1 ps for the low and high fluences, respectively. h) Comparison of the normalized dynamics of the temperature, Raman frequency Δω_*S*_, and linewidth ΔΓ_*S*_ of the Stokes scattering for the low fluence case. The three signals have a similar rise while their decay times are strikingly different, indicating the contribution of nonthermal mechanisms to the frequency and linewidth decays of the TO/LO phonon mode. In all plots, the estimated error is represented by the shading.

To perform a more quantitative analysis of the dynamics of the spectral features, the S and AS Raman spectra are fitted with a Gaussian profile in a spectral region of ± 260–340 cm^−1^, from which the peak intensity, Raman frequency, and linewidth, i.e., full width at half maximum (FWHM), are extracted. The fitted peak intensity, change in phonon frequency (Δω), and change in linewidth (ΔΓ) of both S and AS Raman scattering are shown in **Figure** **2**, where the shading corresponds to the estimated experimental error based on the standard deviation of all spectra acquired prior to the pump excitation as explained in Section S1.5, Supporting Information.

The temporal evolution of the intensities of the S and AS peaks is shown in Figure 2a and b, respectively. An intensity rise reaches its maximum at ≈4 ps, after which the intensity decays and fully returns to equilibrium values after ≈10 ps. In contrast, Δω(*t*) of both S and AS, shows completely different dynamics than that of the intensity (see Figure 2c,d, respectively). The rise in its absolute value, |Δω|, still follows dynamics similar to that of the intensity, which also takes ≈4 ps to reach its maximum value for both bands. Nevertheless, the most notable difference is its slower decay, where Δω(*t*) does not fully decay back to its equilibrium value within the experimental temporal window (200 ps). Figure 2e,f show the dynamics of ΔΓ, for S and AS Raman scattering, respectively. Similarly to the intensity and Raman shift, ΔΓ also reaches its maximum value of approximately ≈1.5 cm^−1^ in ≈4 ps for both bands. However, its decay is strikingly different from that of both the intensity and the Δω(*t*). It is worth noticing that measuring a change in linewidth in ultrafast experiments is extremely challenging since the frequency resolution is compromised by the short pulse duration of the probe pulse. As described in Section S1, Supporting Information, we use a pulse shaper to temporally stretch the probe pulse duration and, therefore, increase the frequency resolution to 14 cm^−1^. This resolution is high enough to deconvolve the contribution of the gaussian laser from that of the Raman phonon mode, in such a way that we can reliably monitor the evolution of the linewidth.

The S and AS peak intensities provide valuable information, as one can calculate the TO/LO phonon temperature from their ratio (see Section S1.3, Supporting Information, for detailed explanation). Figure 2g shows the temperature evolution of the TO/LO Ge phonon mode, before and after excitation, for low (1.5 · 10^20^ cm^−3^) and high pump fluences (4.4 · 10^20^ cm^−3^). The shading represents the temperature uncertainty that we estimated by performing the error propagation of the experimental parameters used to calculate the temperature (see Section S1.5, Supporting Information, for more details). For both cases, the temperature at negative times, i.e., before pump excitation, is slightly above room temperature, indicating a 20 K heating caused by the probe pulse during our measurements. The fluence of the probe is kept as low as possible to minimize this effect, while still providing a sufficiently large signal‐to‐noise ratio. After pump excitation, there is an increase in temperature following the dynamics of the AS intensity, reaching its maximum at ≈4 ps, and subsequently decaying back to equilibrium with a decay time of 2.7 ± 0.2 ps and 2.3 ± 0.1 ps for low and high fluences, respectively, extracted by fitting the decay using a single exponential decay function for both curves. Interestingly, we observe a slightly faster decay as we increase fluence, possibly indicating the presence of an additional energy dissipation channel for optical phonons at larger fluences, i.e., phonon reabsorption by photoexcited carriers.^[^ ^16^, ^17^ ^]^ Figure 2h shows the comparison between the normalized temperature, phonon frequency and linewidth, showing noticeable different decay dynamics.

**Figure 3:**
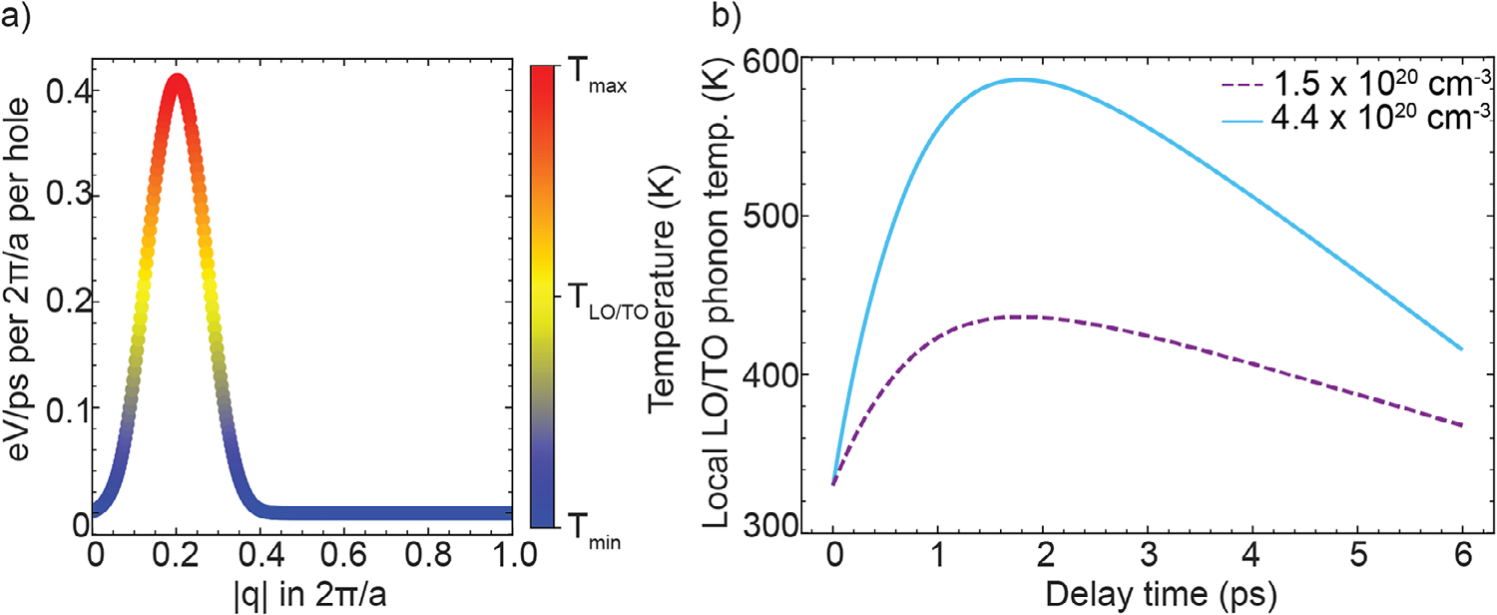
Dynamics of temperature rise of TO/LO modes calculated with time‐dependent two‐temperature model for carriers and phonons, coupled with DFT‐based description of the electron‐phonon and phonon‐phonon coupling in Ge (See Section S2, Supporting Information). a) Hole‐phonon energy transfer rate calculated with DFT as a function of phonon wavevector (|**q**|‐dependent spectral function, see Section S2.2, Supporting Information), which illustrates the localization in the Brillouin zone of the overheated optical modes. b) Calculated local temperature rise of Raman optical modes due to hole‐phonon interaction, for hole densities of 1.5 · 10^20^ cm^−3^ (dashed purple line) and 4.4 · 10^20^ cm^−3^ (light blue solid line). Subsequent decay into acoustic phonons is modeled with a calculated phonon–phonon decay rate of 2.5 ps (see text and Section S2, Supporting Information).

To understand the mechanisms behind the rise and subsequent decay of the TO/LO Raman‐active phonon population and the temperature, we perform DFT calculations of the carrier‐phonon and phonon‐phonon interactions. The timescale of the temperature is determined by the relaxation dynamics of photoexcited carriers. According to our calculations, this rise is attributed to the photoexcited hole dynamics: the dominant scattering process for photoexcited holes is intravalley scattering with optical phonons, which leads to the heating of Raman‐active optical modes (see Section S2, Supporting Information, for full details of the calculations). In contrast, the dynamics of photoexcited electrons is mostly determined by the intervalley scattering^[^ ^10^ ^]^ (see Supplementary Section S2.3), which explains why photoexcited electrons do not influence the dynamics of Raman‐active optical modes. As seen in **Figure** **3**a, photoexcited holes transfer their energy only to phonons localized close to the Brillouin zone center, producing a strong rise in the local phonon temperature. On the other hand, Figure 3b shows our calculated phonon temperature dynamics. According to our DFT calculations, the observed decay times of 2.7 ± 0.2 and 2.3 ± 0.1 ps for high and low fluence, respectively, correspond to the decay of optical modes into acoustic modes via anharmonic coupling. Indeed, the lifetime of Γ‐point optical phonons, calculated within the Relaxation Time Approximation and considering third‐order anharmonic processes, is found to be between 2 and 2.5 ps at room temperature (depending on what DFT and Boltzmann transport equation (BTE) solvers were used; see Section S2, Supporting Information), which is in very good agreement with the measured values. Interestingly, the decay of optical modes into acoustic modes determined both experimentally and theoretically in our work is found to be faster than the one previously reported by Young et al. (≈4 ps at room temperature^[^ ^14^ ^]^). However, it is worth noting that the temporal resolution of Young et al.^[^ ^14^ ^]^ was limited by their pulse duration, 4 ps, which makes it challenging to observe any faster phenomena. Moreover, our measured and calculated lifetime is in agreement with Mendez et al.^[^ ^25^ ^]^ who reported a broadening of 2 cm^−1^ for pristine Ge at 300 K, which corresponds to the lifetime of 2.5 ps.

Besides the S and AS intensity ratio, one can also extract temperature from the Raman frequency shift or the linewidth.^[^ ^26^ ^]^ Interestingly, Figure 2h shows that the temperature rise calculated from the intensity ratio, Δω and ΔΓ, are in very good agreement, which indicates that the contribution to the rise of the Raman frequency and linewidth is given exclusively by the increase of the phonon temperature. Indeed, the increase in temperature Δ*T* is about 150 K at ≈4 ps, which corresponds to a Δω and ΔΓ of 2.4 cm^−1^ and 1.5 cm^−1^, respectively, as shown in Figure 2c–f. These values are in excellent agreement with measurements from conventional Raman thermometry reported in previous works.^[^ ^27^, ^28^ ^]^ On the other hand, Figure 2h shows that the normalized temperature, Raman shift, and linewidth have remarkably different decays, indicating that alongside with a decay in temperature, there are additional contributions to the decay dynamics of the Raman frequency and the linewidth. Particularly, we extract a temperature decay time (2.7 ± 0.2 ps), that is drastically different from that of Δω (44.5 ± 3.7 ps) and ΔΓ (19.4 ± 5.2 ps), shown in Table 1. This indicates that there are more contributions to the decay of these magnitudes in addition to the change caused by the decrease in temperature. In general, the thermal contribution to the Raman shift is influenced by phonon‐phonon scattering, i.e., anharmonicity, and the thermal expansion of the lattice caused by the change in temperature. However, the linewidth is usually only influenced by anharmonicity.^[^ ^26^ ^]^ To explore what other phenomena can influence both Δω and ΔΓ, we performed TR experiments under the same conditions, as well as MD simulations.

**Table 1:** Decay constants, τ, extracted from a single exponential decay fit of the different spectral features, namely: the temperature extracted from the ratio between the anti‐Stokes and Stokes intensities, *T*; the change in Raman shift, Δω, and the change in linewidth, ΔΓ, of the Stokes band together with the damping decay time of the Brillouin oscillations. Error is extracted from the fit.

τ_*T*_ (*ps*)	τ_Δω(*Stokes*)_(*ps*)	τ_ΔΓ(*Stokes*)_(*ps*)	τ_*oscillation*_ (*ps*)
2.7 ± 0.2	44.5 ± 3.7	19.4 ± 5.2	18.1 ± 3.0

**Figure 4:**
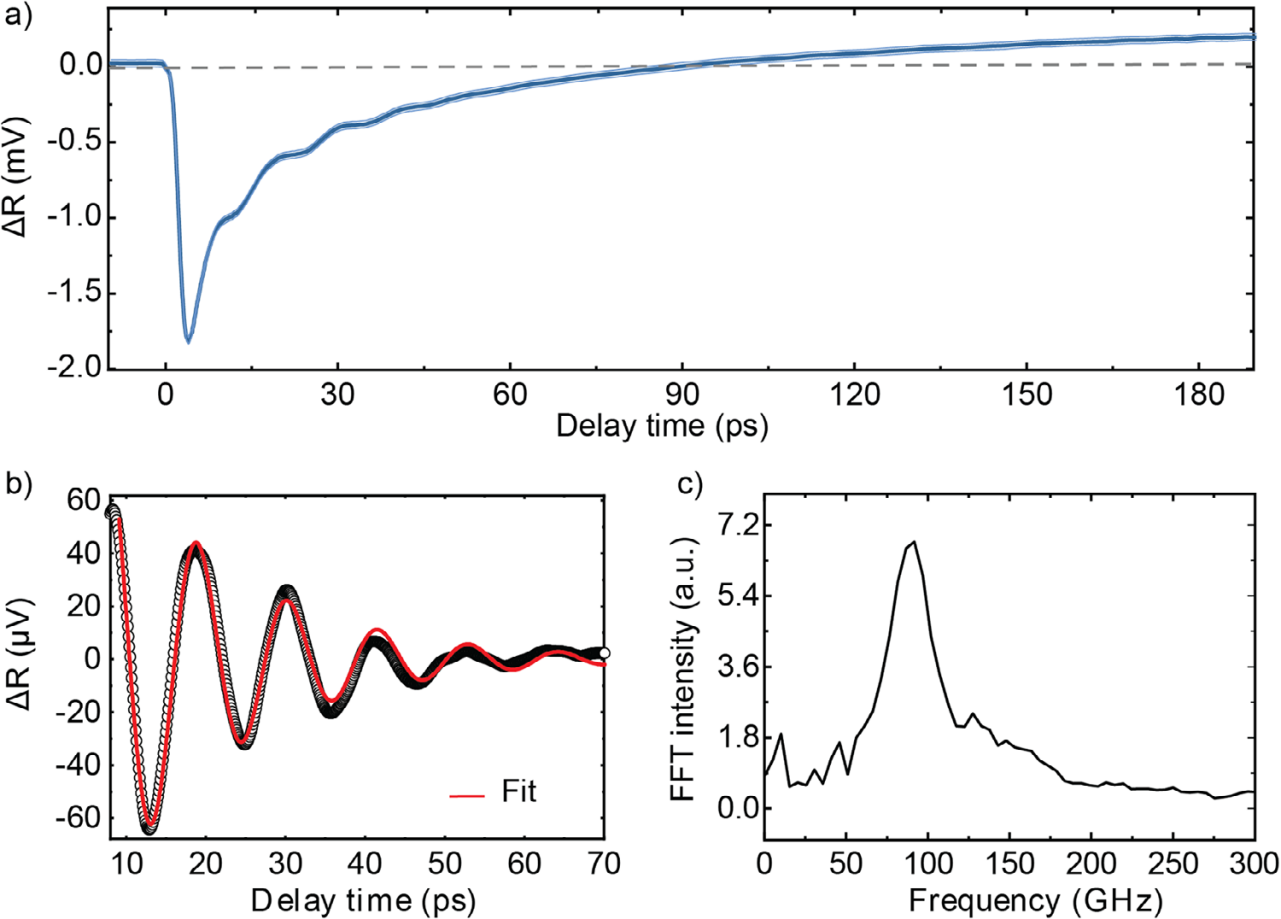
Transient reflectivity on Ge. a) Change in the reflectivity signal of bulk Ge as a function of time for an excitation of 1.4 · 10^20^ cm^−3^, i.e., in the same conditions as the measurements performed by TRRS. The signal shows a sharp decrease in reflectivity after excitation, followed by a recovery, and eventually increases above positive values. b) Oscillating component of the signal after the subtraction of a tri‐exponential decay following the initial decrease in reflectivity (empty circles) and its corresponding fit to a damped oscillating function (solid red line). c) Frequency spectrum calculated by taking the Fourier transform of the oscillating signal.

The pump‐probe setup used to perform TRRS can be adapted to measure TR under the same exact low fluence conditions (see Experimental Section and Section S1, Supporting Information, for further details of the setup). **Figure** **4**a shows the change in reflectivity as a function of time after pump excitation of bulk germanium oriented along the 〈111〉 axis. This signal is related to the change in the material's dielectric constant, ε, caused by the carrier dynamics triggered after excitation. Among the factors that modify ε, there are two main contributors that can affect ε in opposite ways, namely the charge density and the lattice expansion.^[^ ^29^ ^]^

The rise of the TR signal occurs approximately up to 4 ps, as in the case of the Raman spectral properties shown in Figure 2. After this rise, there is a subsequent decay, which we can fit to a tri‐exponential decay with time constants of 2.4 ± 0.6, 11.2 ± 3.6, and 68 ± 14 ps. The contributions involved to the dynamics cancel each other out and eventually make the TR signal rise from negative toward positive values, as shown by the horizontal dashed line in Figure 4a. This is likely the result of two competing contributions. Initially, as the charge carriers population grows and then decays, they hinder the reflectivity signal, while in a second phase, the lattice contribution dominates with a positive contribution to the TR signal, flipping the signal.

Noteworthy, the subtraction of the decay dynamics reveals a strong oscillating signal (see Figure 4b). We fit this signal with a damped oscillating function, from which we calculate a damping time constant of 18.1 ± 3.0 ps and an oscillation period of 11 ± 1 ps. This period corresponds to an oscillation frequency of 91.4 ± 1 GHz as confirmed by the Fourier transform of this oscillating signal, whose frequency spectrum is shown in Figure 4c.

The origin of these oscillations lies in the generation of a coherent acoustic phonon wave packet (acoustic strain field) by the pump pulse, which then propagates through the sample.^[^ ^30^, ^31^ ^]^ We identify this signal modulation as Brillouin oscillations, which arise from the interaction between the light and the generated strain pulse traveling through the sample (see Section S1.6, Supporting Information, for more details).^[^ ^32^ ^]^ Interestingly, the damping time constant of the oscillation is in agreement with the decay time of the linewidth measured by TRRS. This indicates that the strain pulse triggered by ultrafast excitation also influences the dynamics of the phonon linewidth. These coherent acoustic phonons are usually damped over time as phonons lose coherence, due to coupling with other particles, anharmonicity, or defects. Indeed, the Raman linewidth is a measurement of these phenomena, as the peak broadening is related to the phonon lifetime and damping mechanisms. The correlation between the time evolution of this broadening and the damping time of the observed Brillouin oscillations is a unique and powerful tool to determine the underlying physical mechanism governing our observations. It is worth highlighting that we report for the first time this unique correlation as measuring the time evolution of the linewidth requires a proper balance between temporal and frequency resolutions that we achieved by tuning the properties of the probe pulse, as explained in Section S1, Supporting Information.

### 2.2 Influence of Strain on Phonon Dynamics

**Figure 5:**
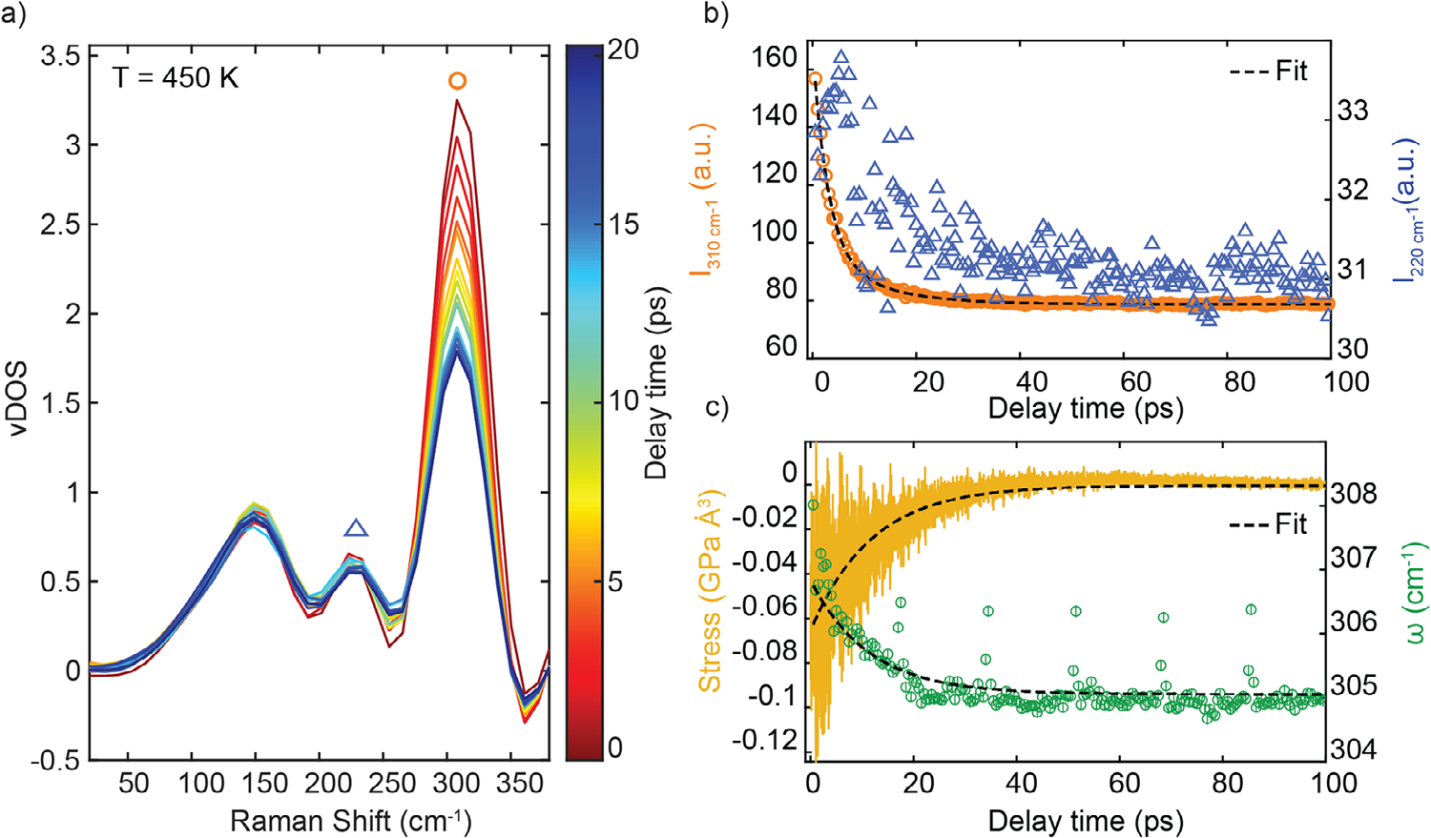
Molecular dynamics results. a) VDOS at different delay times of the central region of the computational cell. The excess energy initially injected into modes around 310 cm^−1^ (red curve) progressively decays and at sufficiently long times, the equilibrium VDOS is recovered (blue curve). b) Computed intensity of the 310 cm^−1^ peak marked with an orange dot in panel a (orange symbols, left y‐axis; bi‐exponential decay fit to the data as black dashed line) and of the 220 cm^−1^ peak marked with a blue triangle in panel a (blue triangles, right y‐axis) of the VDOS as a function of time. c) Frequency of the optical mode (green circles, right y‐axis) and stress (yellow line, left y‐axis) as a function of time, together with the corresponding fitted exponential trend (single exponential) with τ_ω_ = 11.3 ps and τ_σ_ = 11.8 ps. The excitation temperature is *T*_exc_ = 450 K (see Figures S10, S11, and S14 (Supporting Information) in Section S3.3 (Supporting Information) for data corresponding to *T*_exc_ = 600 K).

To understand more about the effect of strain on the phonon dynamics, we performed MD computational experiments of bulk Ge in a computational cell (*L* = 85.1 nm) that we partitioned into three regions (see Section S3, Supporting Information, for full details of the calculations). After equilibrating the system at 300 K, we used a Generalized Langevin Thermostat (GLE)^[^ ^33^, ^34^, ^35^ ^]^ that selectively pumps energy in the central region (2.8 nm‐long) only on vibrational modes within a frequency window Δω = 0.5 cm^−1^ centered around ω_0_ = 310 cm^−1^. This selective thermalization of atomic degrees of freedom mimics the experimental conditions, where the laser injects energy into specific modes (*pump*). We maintain this nonequilibrium condition for 500 fs, realizing two different excitation conditions at *T*_exc_ = 450  and 600 K –corresponding to the experimental maximum temperatures measured for the two explored fluences– and then remove any temperature constraint, letting the system free to reach again the thermodynamic equilibrium at 300 K. We monitor the decay of the phonon excited state by computing the time‐dependent vibration density of states (vDOS) (see Section S3, Supporting Information, for more details). In **Figure** **5**a, we show how the excess energy results in an overpopulated optical peak of the vibrational spectrum, located at around ω_0_ = 310 cm^−1^, with decreasing intensity as a function of time. In fact, the energy, initially placed in the optical phonon modes, quickly decays following a two‐times exponential decay with decay constants τ_*I*1_ = 2.93 ± 0.07 ps and τ_*I*2_ = 11.4 ± 1.1 ps when exciting the system at 450 K (τ_*I*1_ = 2.82 ± 0.04 ps and τ_*I*2_ = 11.7 ± 1.1 ps for 600 K, see Figure S9, Supporting Information). It is worth highlighting that instead the experimental data could be fitted with a single exponential decay. We understand this difference considering that TRRS probes the dynamics of specific phonon modes, while the MD computational experiments can also capture further changes in the local temperature and strain affecting the VDOS.

Our simulation suggests that this decay is associated with the excess of energy being transferred to the lower frequency acoustic modes through phonon–phonon scattering, in agreement with our DFT calculations. As shown in Figure 5b, while the optical peak decays (orange circles, left y‐axis), the peak at ω ≈ 220 cm^−1^ (blue triangles, right y‐axis) is characterized by a fast rise and a slower decay. A similar qualitative behavior is observed for the acoustic peak centered around 150 cm^−1^, see Figures S12 and S13, Supporting Information. The low frequency phonons rise time (of about 3 ps) matches the decay time of the optical one, clearly indicating anharmonicity as the main energy dissipation mechanism for the cooling of the TO/LO phonon mode.

We also compute the time evolution of the frequency shift, which shows a single time constant of τ_ω_ = 11.3 ± 0.4 ps for an excitation of 450 K (Figure 5c, right y‐axis). The observed dynamics is the combination of the phonon thermalization, i.e., the decay of optical modes into acoustic modes, occurring within a few ps, and the following thermal diffusion. The atoms in the local area excited by the pump pulse decay to a final ground state which is hotter than the equilibrium one.^[^ ^36^ ^]^ The resulting temperature gradient will drive the spatial diffusion (see temperature difference between excited and unexcited regions in Figure S9, Supporting Information). The temperature difference decays with a double exponential as well, with a fast relaxation of 2.96 ± 0.03 ps and a slower time constant of 11.52 ± 0.03 ps for *T*_exc_ = 450 K, which confirms what is observed for the peak intensities in Figure 5b. These slower components, including the tail of the intensities and the frequency, can be traced back to the contribution of the stress induced by the *pump* signal and the gradual adjustment of the lattice spacing. Initially, the rapid anharmonic decay redistributes energy among modes, but the slower adjustment of the average bond distances (akin to thermal expansion) lags, leading to a more gradual frequency shift. Indeed, we calculated the atomic stress difference between the excited and unexcited regions (Figure 5c, left y‐axis), finding rise times of 11.8 ± 0.2 ps (11.7 ± 0.3 ps), which is consistent with τ_ω_ and with the longer component of the intensity decay, τ_*I*2_. These results suggest that the slower behavior observed for the Raman frequency shift could also depend on the local thermal‐strain relaxation: in fact, the optical phonon frequency shifts with volume (or equivalently, stress) according to its mode Grüneisen parameter γ: Δω/ω_0_ = −γΔ*V*/*V*. The excess energy delivered by the pump pulse will result in a locally expanded region, causing a red shift of the excited optical mode. Finally, we also extracted the FWHM temporal evolution of the excited peak from our MD simulations (see Figure S11e,f, Supporting Information), and found it to exhibit an intermediate dynamical profile that qualitatively matches our TRRS measurements. We argue that this behavior arises from two competing processes. Following excitation, the system is highly anharmonic, resulting in a large FWHM. As phonon–phonon scattering drives thermalization of the excited mode, this anharmonicity rapidly diminishes, yielding a fast reduction in linewidth. At the same time, energy transfer from the pumped mode into other vibrational modes heats the local lattice environment. This higher local temperature sustains an enhanced scattering rate, which in turn continues to broaden the Raman peak. Thus, although the intrinsic anharmonic broadening decays quickly, the residual thermal bath remains ‘hot’ on longer timescales, maintaining the FWHM at an intermediate value until the excess heat diffuses away. Details of the computational protocol, the temperature, intensity, and stress time evolution, and convergence tests on the computational cell size can be found in Section S3, Supporting Information.

## 3 Discussion

TRRS and TR access the energy flow after an ultrafast excitation in (111)‐oriented bulk Ge. We observe an increase in the TO/LO phonon temperature, which is given by the transferring of energy from photoexcited holes following ultrafast excitation, as proved by DFT calculations. We calculate a subsequent temperature decay time of 2.7 ps, which we found to be in excellent agreement with both DFT and MD simulations, indicating an anharmonic TO/LO phonon decay into acoustic phonons as the main energy dissipation mechanism. Remarkably, the temperature decay time is strikingly different from that of the change in Raman frequency and the change in linewidth, which we attribute to time‐evolving thermal strain, as proved by MD simulations. In addition, we observe Brillouin oscillations from our TR data performed under the same conditions as TRRS. This reflectivity modulation is generated from the interaction of the probe beam and a longitudinal strain pulse launched by the pump laser, which subsequently travels through Ge. Interestingly, the decoherence time of these oscillations, 18.1 ps, nicely matches the decay time of the Raman linewidth. This is indicative of a correlation between the coherent acoustic phonons launched by the ultrafast laser and the TO/LO phonon mode dynamics. Therefore, our work highlights physical mechanisms that occur on different timescales: the temperature decay shows the fastest process with a decay time of 2.7 ps, which corresponds to the anharmonic decay. The analysis of the Raman peak broadening shows an intermediate regime, with a decay time of 19.4 ps, which we correlate to the decay time constant of the Brillouin oscillations, 18.1 ps, that are triggered by the pump excitation. Finally, the slowest timescale is observed from the temporal evolution of the phonon frequency, whose decay time is 44.5 ps, and we link it to stress induced by the pump excitation. These results offer a unique understanding of the energy dissipation on ultrafast time scales and the entanglement between fundamental excitations and pump‐induced strain, critical for the improvement of micro‐ to nanoelectronic devices. Moreover, our findings are of highly significance for the semiconductor industry, since relevant process such as switching speeds, optical response times or efficiencies are directly determined by the interaction of charge carriers and phonons at their characteristic timescales. Understanding these processes is crucial for the optimization of materials and device architectures.

## 4 Experimental Section

### Germanium Bulk Sample

The germanium sample was a piece from a commercial wafer both from the company University Wafer. Crystal direction (111), single side polished (SSP), prime quality, float zone, undoped with a resistivity > 50 Ohm·cm^−1^.

### Ultrafast Spectroscopy

TR and TRRS, were performed at room temperature on (111)‐oriented intrinsic crystalline Ge, which was excited with a pump pulse with a duration of 30 fs and a wavelength of 800 nm (1.55 eV). The response of the sample was monitored using a less intense probe pulse with a duration of 1.30 ps and a wavelength of 640 nm, which was focused on the sample with a 20x objective that provides a spot size of 3 μm, while the pump beam size was 5 μm. The probe fluence was set at ⩽ 0.3 mJ·cm^−2^ while the pump fluence is varied in a range of 2–9 mJ·cm^−2^. The pump pulse goes through a mechanical delay line, allowing for a controlled delay time between the pump and the probe within a temporal window of 200 ps. The pump and probe pulses were in a cross‐polarization configuration. A polarization parallel was selected to the probe polarization in the detected signal, making it easier to filter out the pump light. Moreover, a shortpass spectral filter with a cutoff frequency of 700 nm was also used to filter any remaining pump intensity. The probe polarization was parallel to the (010) crystal direction. According to the Raman selection rules,^[^ ^37^ ^]^ this orientation allows to probe 2/3 of the TO and 1/3 of the LO phonon modes, which are degenerated at the zone center, and are collectively referred as TO/LO phonon mode. For each delay time, four spectra were collected and averaged with a triple‐spectrometer with a 300 s integration time. For TR measurements, changes in the sample reflectivity were monitored as a function of time delay using a balanced photodiode combined with a lock‐in amplifier detection scheme.

### Temperature Calculation

The temperature was calculated from the ratio between the AS and S intensity as

(1) $$\frac{I_{AS}}{I_{S}}=C_{exp}{\left(\frac{\omega_{exc}+\omega }{\omega_{exc}-\omega }\right)}^{4}e^{-\frac{\hbar \omega }{k_{B}T}}$$

where ω is the frequency of the probed phonon, *T* is the temperature, ω_*exc*_ is the excitation frequency, and *C*_*exp*_ is an empirically‐determined constant related to the experimental differences in the light collection between S and AS spectral windows given by the different instrument response (see Section S1.4, Supporting Information, for more details).

### Coupled Hot Hole and Phonon Dynamics Simulations

The hot hole dynamics was described by propagating in time the coupled time‐dependent Boltzmann transport equations for carriers and phonons, in the framework of excess‐energy dependent model which was described in Ref. [10] (see Section S2, Supporting Information, for details). This method included carrier‐phonon and phonon–phonon collision terms, and a single effective LO/TO phonon mode. In the case of thermalized distributions considered in this work,^[^ ^38^ ^]^ this approach is equivalent to two‐temperature model.^[^ ^39^ ^]^ Excess‐energy dependent carrier‐phonon collision term was modeled based on DFT calculations (see below), while phonon‐phonon collision term was described by a constant decay rate based on DFT result.

Coming to carrier distribution function, energy and band structure considerations for the single‐photon absorption of 1.55eV photons in Ge yield holes temperature of around 1500 K.^[^ ^38^ ^]^ However, according to Ref. [40], intraband absorption plays an important role for holes in Ge, provided that photoexcited carrier densities are relatively high as in the experiment. While having negligible effect on the estimation of the photoexited carrier densities, intraband absorption could lead to important changes in photoexcited hole temperatures. Therefore, in order to account for a few percent of holes which might be concerned by intraband absorption of 1.55 eV photons, the photoexcited hole temperature of 2500 K was considered for the calculation of hole‐phonon energy transfer which lead to temperature rise of LO/TO phonons.

Finally, coming to ab initio calculations, Ge was described using DFT within the LDA approximation, with lattice parameter of 10.696 a.u..^[^ ^41^ ^]^ The rate of energy transfer from carriers to phonons was calculated by DFT‐based approach using quantum espresso,^[^ ^42^ ^]^ Wannier90^[^ ^43^ ^]^ and EPW^[^ ^44^ ^]^ codes (see Section S2, Supporting Information for details). The rate of energy transfer from carriers to phonons changes with excess energy as the density of final states. Because of this, the calculated ab initio data could be successfully modeled with energy‐dependent model of the form $C_{el-ph}\left(1-f\left(\varepsilon \right)\right)\sqrt{\varepsilon }$, where ϵ was excess energy, *f*(ϵ) was hole distribution and *C*_*el* − *ph*_ was a constant (see Section S2, Supporting Information). For the calculation of LO/TO phonon decay via phonon–phonon interaction, D3Q code^[^ ^45^ ^]^ was used to obtain the three‐phonon anharmonic coupling rate for zone‐center optical phonons.

### Molecular Dynamics Simulations

Pump‐probe molecular dynamics simulations were performed with periodic boundary conditions on a bulk germanium box with length *L* = 85.1 nm and section of 2.8 × 2.8 nm^2^, for a total of 30000 atoms. Germanium atoms were modeled according to Tersoff potential.^[^ ^46^ ^]^ The system was initially prepared at 300 K with an NPT run by means of the Nosé‐Hoover thermostat for 50 ps with a timestep of 1 fs, in order to reach the equilibrium density at 300 K. Then, a NVT run was performed with the stochastic velocity rescaling thermostat^[^ ^47^ ^]^ for further 50 ps with a timestep of 1 fs.

From this initial setup, the system was divided in three distinct regions to model the experimental conditions of a laser hitting a localized spot in the sample. As shown in the schematic representation of Figure S8a (Supporting Information in Section S3.1 Supporting Information, the central area which is 2.8 nm‐long, was excited according to the Generalized Langevin Equation (GLE) thermostat while the surrounding regions were left unperturbed at room temperature. The GLE only acts on the system for 500 fs, realizing an initial excitation of *T*_exc_ = 450 K or 600 K, corresponding to the temperature rise observed in the experiments. After the excitation, the thermostat was turned off and the simulation cell evolved freely in the NVE ensemble toward its final equilibrium configuration. The corresponding temperature profile was reported in Figure S8b,c (Supporting Information) for *T*_exc_ = 450 K and 600K respectively: a *t* = 0, the system is characterized by a hot‐spot, which progressively smooths out during the relaxation. The results reported in this work were obtained by performing a configurational average over 100 statistically independent runs (i.e. initialized with different initial atomic velocities).

## Conflict of Interest

The authors declare no conflict of interest.

## Author Contributions

G.R. and B.A. contributed equally to the manuscript. I.Z. conceived the experiment and supervised the project. G.R., A.K.S., J.M.S.‐G. and B.A. developed the setup and measurements protocol. G.R. and B.A. performed the time‐resolved Raman and transient reflectivity measurements and analyzed the data. J.S., R.S., and N.V. performed the DFT calculations. R.D., C.M., and R.R. performed the MD simulations. All authors discussed the results and either wrote or reviewed the paper.

## Supporting information

Supporting Information

## Data Availability

All data within the article and the Supplementary Information that support the findings of this study are openly available in ZENODO at https://doi.org/10.5281/zenodo.17593967.
