# Supplementary material for: Unraveling Energy Flow Mechanisms in Semiconductors by Ultrafast Spectroscopy: Germanium as a Case Study
Source: Adv Sci (Weinh). 2026 Jan 12;13(11):e15470. doi: 10.1002/advs.202515470 (PMC12931156; doi:10.1002/advs.202515470)
Supplement: Supplementary file 1 — Supporting Information [file ADVS-13-e15470-s001.pdf]

# Supporting Information – Unraveling Energy Flow Mechanisms in Semiconductors by Ultrafast Spectroscopy: Germanium as a Case Study

*Grazia Raciti*[1] *Begoña Abad*[1] * *Riccardo Dettori* *Raja Sen* *Aswathi K. Sivan* *Jose M. Sojo-Gordillo* *Nathalie Vast* *Riccardo Rurali* *Claudio Melis* *Jelena Sjakste* *Ilaria Zardo* *

Dr. G. Raciti, Dr. B. Abad, Dr. Aswathi K. Sivan, Dr. J. M. Sojo-Gordillo, Prof. I. Zardo
Department of Physics, University of Basel, Basel, 4056, Switzerland
Email Address: b.abad@unibas.ch, ilaria.zardo@unibas.ch
Prof. I. Zardo
Swiss Nanoscience Institute, University of Basel, Basel, 4056, Switzerland
Dr. R. Dettori, Prof. C. Melis
Department of Physics, University of Cagliari, Monserrato, CA 09042, Italy
Dr. R. Sen
SATIE, CNRS, ENS Paris-Saclay, Université Paris-Saclay, Gif-sur-Yvette, 91190, France
Prof. N. Vast, Prof. J. Sjakste
Laboratoire des Solides Irradiés, CEA/DRF/IRAMIS, Ecole Polytechnique, CNRS, Institut Polytechnique de Paris, Palaiseau, 91128, France
Dr. R. Rurali
Institut de Ciència de Materials de Barcelona, ICMAB–CSIC, Campus UAB, Bellaterra, 08193, Spain
[1] These authors contributed equally to this work.

# S1 Experimental details

## S1.1 Multi-scheme pump-probe setup

Figure S1 shows a scheme of the experimental setup used to perform time-resolved Raman spectroscopy (TRRS) or transient reflectivity (TR) measurements, depending on the detection scheme. Both techniques rely on an ultrafast hybrid ytterbium fiber laser from Spectra-Physics (Spirit 1030-70) with a wavelength of 1030 nm, a pulse duration of hundreds of femtoseconds ($<$ 400 fs), and a repetition rate of 1 MHz. The output of this laser is split into pump and probe pulses, which are steered to a non-collinear optical parametric amplifier (NOPA) and an optical parametric amplifier (OPA), respectively, that can tune the wavelength of the ultrafast beams in a range of 600 – 900 nm.

In the case of TRRS measurements, Raman spectra are tracked as a function of delay time between pump and probe. Remarkably, the OPA output beam has a pulse duration of 180 fs and goes directly to a pulse shaper, which stretches the temporal duration by one order of magnitude, up to 1.30 ps. This is crucial to improving the spectral resolution of the Raman measurements, as time and frequency are Fourier conjugates, and the shorter the pulse, the larger its spectral bandwidth. Stretching the pulse enables us to achieve a spectral resolution of 14 $cm^{-1}$, with which we can resolve the different spectral features of the probed Raman spectra. This probe is used to measure Raman spectra at different times before and after pump excitation, acquired by a triple spectrometer (TriVista TR 555) with three 1500 l/mm, 1500 l/mm, and 1800 l/mm gratings. The dispersed light is collected with a Princeton Instrument charge-coupled device (CCD) (ProEM+).

For TR measurements, the change in reflectivity of the sample surface is tracked as a function of the delay time between the pump and probe pulses. TR uses modulation of the pump laser by a chopper whose frequency is locked to 666 Hz and is used as a reference for a lock-in amplifier (SR830). This approach enables filtering of the noise at various frequencies and amplifying the signal that is detected by a balanced photodiode (Physics Basel, SP 1'023).

While the selection of the pump-probe temporal overlap ($t_0$) in standalone TR experiments has little transcendence, in this work, $t_0$ is consistently used as the temporal reference for our TRRS experiment as well. This ensures a perfect synchronization between the TR signal and the dynamics observed by TRRS. Here, $t_0$ is defined as the time in which the TR signal starts to rise, since the reflectivity response immediately changes with the creation of charges by the pump pulse excitation, its temporal resolution is given by the cross-correlation in between the pump and probe pulses, whose durations are 30 fs and 1.30 ps, respectively.

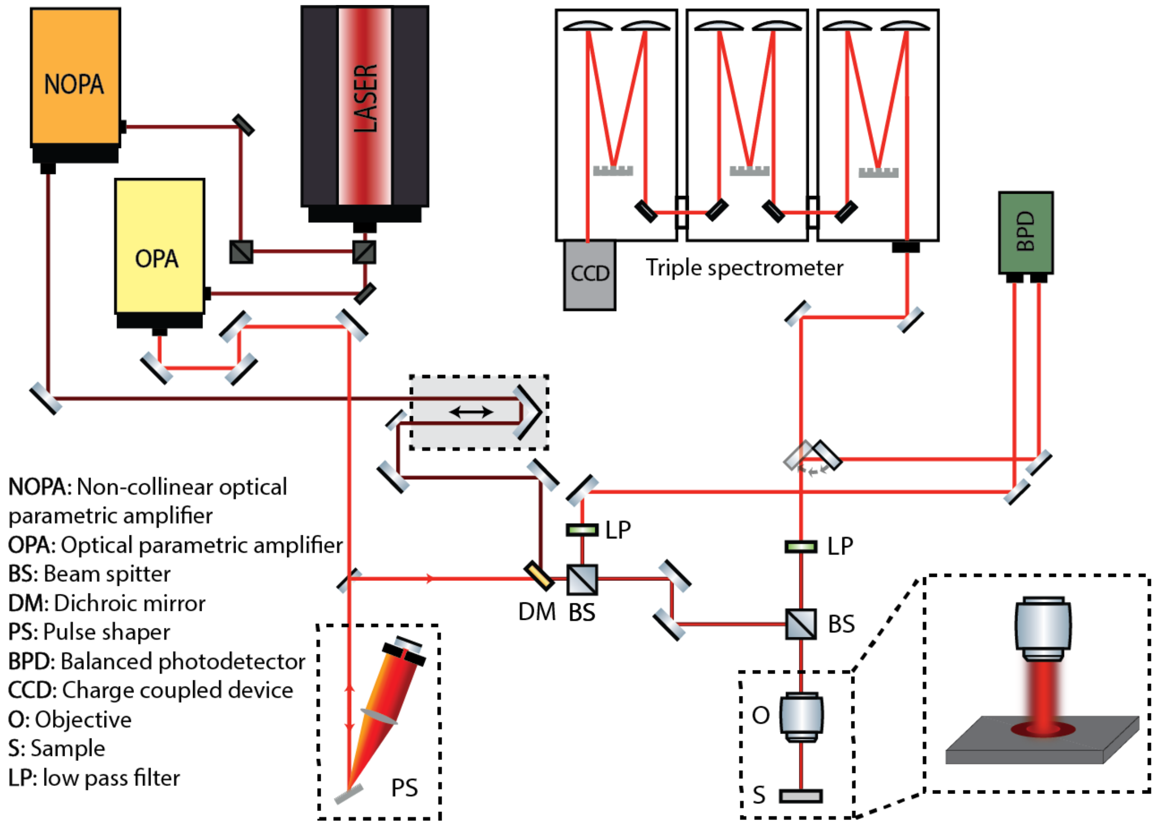


Figure S1: **Time-resolved Raman and transient reflectivity pump-probe setup**. A femtosecond pulse laser (1040 nm) is split into two beams that are steered to optical parametric amplifiers and that will act as a pump and probe. A pulse shaper (PS) is used to stretch the pulse up to 1.30 ps in order to increase the spectral resolution ($14\,\mathrm{cm}^{-1}$) for Raman spectroscopy measurements. Both beams are shined on the sample by using a 20x objective. The backscattered light is then collected and analyzed either through a triple spectrometer (TRRS) or a balanced photodiode (TR).

The photoexcited carrier density induced by the pump pulse, N, was estimated using the following relation[1]

$$N = (1 - R)\frac{F\alpha}{h\nu} \tag{1}$$

where F is the fluence ($J/cm^2$), R is the sample reflectance, $\alpha$ is the optical absorption coefficient ($cm^{-1}$), and $h\nu$ is the photon energy of the pump laser ($J$). The fluence is calculated using the following expression

$$F = \frac{P_{avg}}{f_{rep}A} \tag{2}$$

Where $P_{avg}$ is the average power ($W$) measured by a power meter, $f_{rep}$ is the laser repetition rate ($Hz$), and $A$ is the laser beam spot area ($cm^2$). The latter is calculated from the beam size that is measured using the knife-edge method[2], performed in the same conditions in which the experiments are carried out.

## S1.2 TRRS signal processing

The collection of the data is automated with a self-written Python script. Starting from the anti-Stokes side, a spectrum per time-step of the delay line is collected. Before moving the spectrometer grating to the Stokes side, we acquire a neon lamp spectrum, which, as explained afterwards, serves to calibrate the spectral window. We then proceed with the acquisition of the Stokes spectra using the same conditions as for the AS. After this, the neon lamp spectrum is acquired again to calibrate the Stokes spectral window. When the measurement is completed, two sets of data, one for S and the other for AS, as a function of the delay time between the pump and the probe, are generated. Absolute frequency calibration of the Raman spectra was done by using the well-known Neon lamp emission lines, recorded at the end of each dataset, as reference points to correct the spectral axis. All the data analysis is automated with a self-written MATLAB script. In the following, we describe the data analysis steps for a representative spectrum; the same procedure was then applied to the entire dataset. A representative raw Ge Raman spectrum is plotted in Figure S2a. It exhibits a peak at $\approx 300\,\mathrm{cm}^{-1}$, which is the transverse optical (TO)/longitudinal optical (LO) degenerate phonon mode in bulk Ge. First, we use a linear fit to account for the background signal in the spectrum. The red points in Figure S2b represent the selected points used to determine the linear fit of the background, which is shown with the red solid line. This linear fit is then subtracted from the data. The resulting spectrum displayed in Figure S2c is then fitted using a Gaussian function given by:

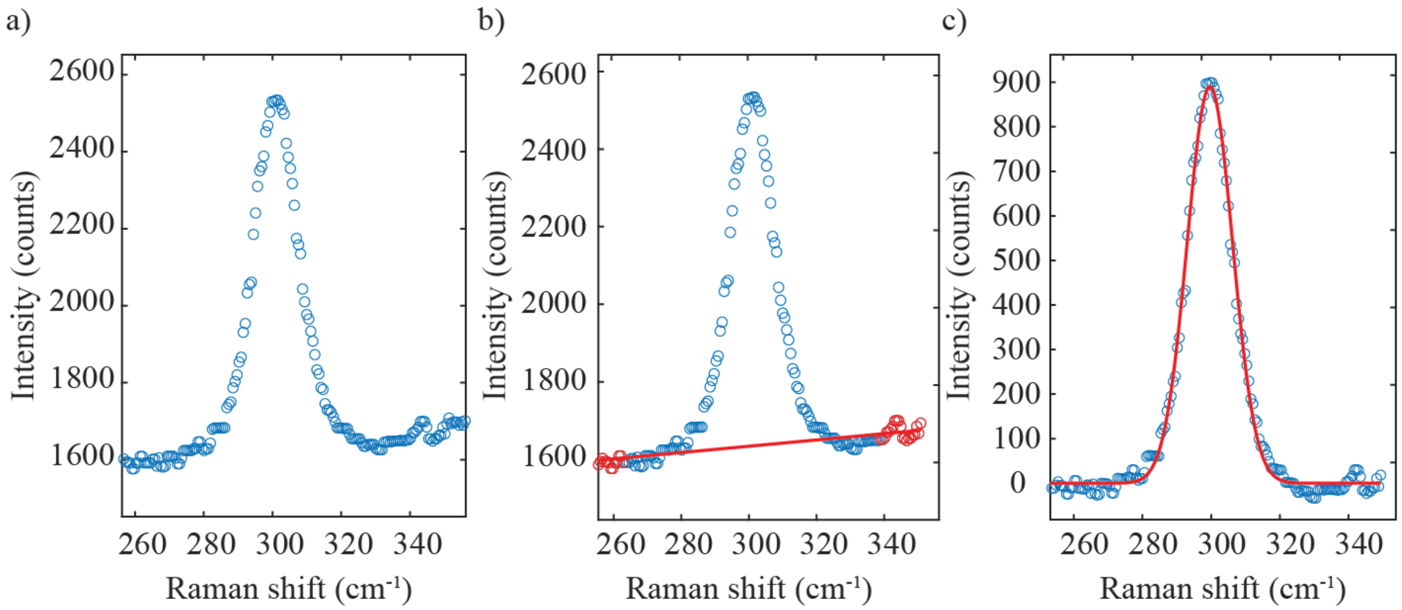


Figure S2: a) Representative raw Ge Raman spectrum. b) Linear fit of the baseline to remove the background signal. The red data points are the ones selected to perform the linear fit represented by the solid red line. c) Raman spectrum after the baseline subtraction. The red solid line represents the Gaussian fit.

$$y_G = y_0 + \frac{A}{w\sqrt{\pi/2}} e^{\frac{-2(x-x_0)^2}{w^2}} \tag{3}$$

where $y_0$, $x_0$, $A$, $w$ represents offset, center, linewidth and area respectively. We use a Gaussian function because the Gaussian spectral lineshape of our probe pulse dominates over the Lorentzian lineshape of the phonon mode, as the FWHM at room temperature is $13\,\mathrm{cm}^{-1}$ for the probe pulse and $2.5\,\mathrm{cm}^{-1}$ for the phonon mode[3;4]. The intensity of the peak is calculated from the fit parameters as follows:

$$I = y_0 + \frac{A}{w\sqrt{\pi/2}}. \tag{4}$$

In addition, in Figure 18 we show the difference spectra, which are calculated by subtracting the average intensity of all spectra acquired before excitation from each spectrum at selected times. Moreover, to avoid artifacts in the resulting difference, the dynamic Raman frequency change, $\Delta\omega(t)$, must be taken into account and suppressed. For this purpose, we extract the average Raman shift of all spectra acquired before excitation ($\overline{\omega}(t < 0) = \pm\, 301\,\mathrm{cm}^{-1}$) and subtracted it from the Raman shift at each selected time, $\omega(t)$, obtaining $\Delta\omega(t)$. The difference between these values, $\omega(t) - \Delta\omega(t)$, is the corrected Raman frequency, $\omega_{corr}(t)$, which is extracted from each spectrum displayed in Figure 18c and d.

### S1.3 Experimental phonon temperature calculation

The intensities of the S and AS bands are related to the phonon population, from which the temperature of the phonon mode can be calculated[5]. Indeed, the temperature of the phonon mode can be tracked by measuring the change of any of the spectral properties, Raman frequency, linewidth, or ratio of S and

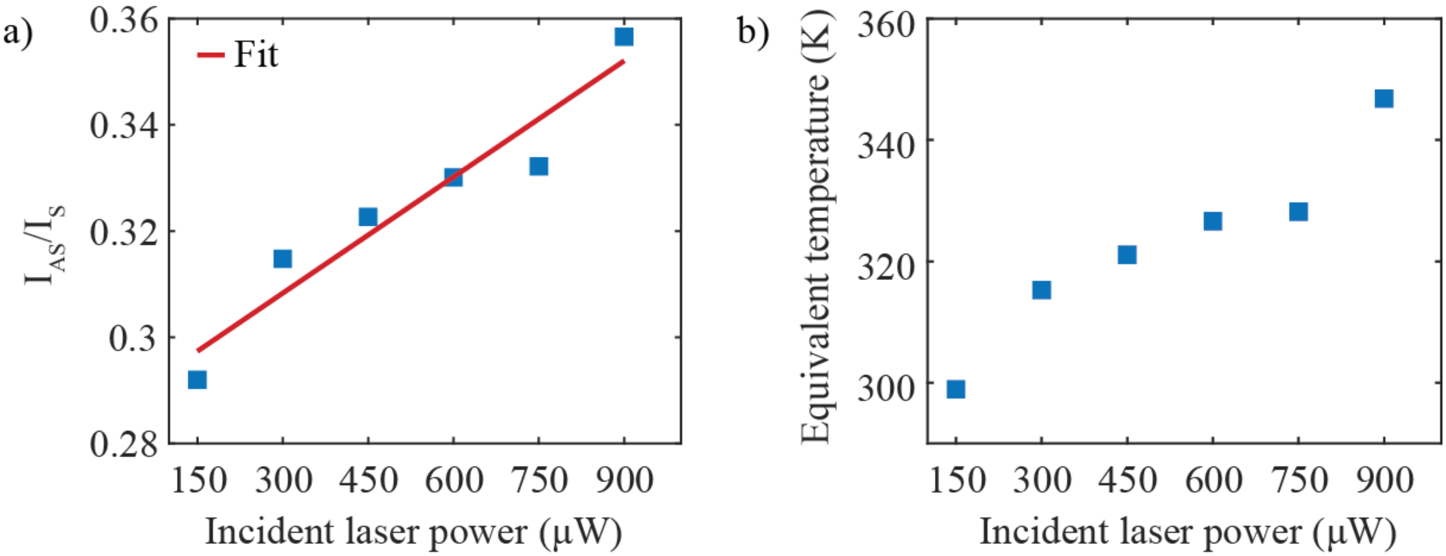


Figure S3: a) Intensity ratio between AS and S signals at each probe laser power (blue squares). The red solid line represents the linear fit used to extract the value of the intensity ratio to the zero heating power, which is needed to determine the calibration factor. b) Equivalent temperature calculated by using the calibration factor and employed to determine the probe power to perform the experiments, avoiding heating effects.

AS intensities. This approach is the well-known Raman thermometry that has been used to extract temperature maps and thermal properties of numerous materials[6]. This technique is traditionally performed under equilibrium conditions using continuous-wave laser sources to probe. While conventional Raman thermometry probes the average temperature of the lattice, ultrafast Raman thermometry measures the temperature of the probed optical phonon mode at a specific time after excitation[5]. Importantly, Raman thermometry relies on a prior calibration, in which the sample is heated up to correlate phonon spectral features such as linewidth and frequency with temperature. This calibration is essential to extract accurate temperature values from spectral changes. However, an alternative approach allows one to extract the temperature from the ratio between the AS and S intensity, as Equation (5) indicates[7], without any prior calibration of the Raman properties as a function of temperature:

$$\frac{I_{\mathrm{AS}}}{I_{\mathrm{S}}} = C_{\mathrm{exp}} \left( \frac{\omega_{\mathrm{exc}} + \omega}{\omega_{\mathrm{exc}} - \omega} \right)^4 e^{-\frac{\hbar\omega}{k_B T}} \tag{5}$$

where $C_{\mathrm{exc}}$ is an empirically-determined constant related to the experimental differences in the light collection between S and AS spectral windows given by the different instrument response at these relatively large separations of frequencies. In this equation, $\omega$ is the frequency of the probed phonon, $T$ is the temperature, and $\omega_{\mathrm{exc}}$ is the excitation frequency. Therefore, we extract the temperature from this intensity ratio after determining $C_{\mathrm{exp}}$ by calibrating our experimental setup, as explained in the next section.

## S1.4 Temperature calibration

To determine $C_{exp}$, we perform a laser probe power study at room temperature (300 K). We perform steady-state Raman spectroscopy using only the probe pulse. We collect different Raman spectra for

both S and AS, varying the incident probe laser power from 150 – 900 $\mu$W. Then, for each laser power, the corresponding intensity ratio is calculated as shown in Figure S3a. We extrapolate the intensity ratio to zero laser heating by using a linear fit function (red solid line in Figure S3a). Plugging this value in Equation (5) we extract a calibration factor of 1.06. We can use this calibration factor to determine the equivalent temperature of the phonon mode at different probe powers. The results are shown in Figure S3b. The increase in temperature with incident laser power indicates that we are heating the sample. In addition, this procedure is useful to determine the power level used in the experiment that minimizes the probe heating of the sample, which could affect the measured dynamics. Therefore, we found a value of 150 $\mu$W for the probe laser to be a good compromise between obtained signal-to-noise ratio and the resulting sample heating.

### S1.5 Uncertainty calculation

In this work, there are three types of errors:

- **Error in the time evolution of intensity, frequency shift and linewidth in TRRS and change in reflectivity in TR:** this error is quantified as the standard deviation of the values taken at times before ultrafast excitation. In TRRS, we could have taken the error of these parameters from the fits of the Raman spectra at each time step. However, this error is considerably smaller than the one given by the standard deviation of the pre-time zero points. Therefore, taking the more conservative error embodies more precisely all the error sources (laser stability, detector noise, etc.). This error is illustrated in the figures as a shading around the data. It is worth noting that this error in the TR measurement, shown in Figure 5 is almost not visible, since the signal-to-noise ratio is very high, so the standard deviation before time zero is very small as compared to the signal.

- **Error in the decay times calculation:** the decay times for the temperature, frequency peak, and linewidth decay times in TRRS are extracted directly from the fit to an exponential function.

- **Error in Brillouin oscillations:** the error of the Brillouin frequency, as well as the damped oscillation decay time in TR is extracted from the fit of the data to a damped oscillating function.

- **Error in temperature:** we calculate the error in the temperature by propagating the errors of the AS and S intensities and the calibration factor $C_{\text{exp}}$, by using the following expression:

$$\sigma_T = \frac{\hbar\omega}{k_B \left[\ln\left(\frac{C_{\text{exp}}(\omega_{\text{exc}}+\omega)^4 I_S}{(\omega_{\text{exc}}-\omega)^4 I_{AS}}\right)\right]^2} \sqrt{\left(\frac{\sigma_{I_{AS}}}{I_{AS}}\right)^2 + \left(\frac{\sigma_{I_S}}{I_S}\right)^2 + \left(\frac{\sigma_{C_{\text{exp}}}}{C_{\text{exp}}}\right)^2}. \tag{6}$$

We reflect these errors as a shading in Figure 2 and Figure 4. The error in the change in frequency and linewidth of the S band is smaller than the AS one, since the S signal is higher because of its larger probability. In contrast, the fitted intensity error is greater for S scattering, since $\Delta I/I$ is larger for AS scattering, as shown in Figure 1c and d.

### S1.6 Brillouin oscillations

We observe low-frequency oscillations ($\sim$ tens of GHz) from our transient reflectivity signal which we identify as Brillouin oscillations[8]. They are detected from the interference between the probe light reflected by the surface and the probe light reflected from the moving strain wave[9]. Indeed, the frequency of the Brillouin oscillations, in the case of normal incidence, is given by:

$$f_{Brillouin} = \frac{2v_L n(\lambda)}{\lambda} \tag{7}$$

where $v_L$ and $n(\lambda)$ are the longitudinal-acoustic sound velocity and the refractive index at the probe wavelength $\lambda$. The longitudinal acoustic sound velocity for Ge is approximately $v_L = 5400$ m/s[10], while the refractive index is 5.4067[10]. The expected Brillouin oscillation at the experimental wavelength of the probe 640 nm has a frequency of $f_{Brillouin} = 91.13$ GHz, in excellent agreement with what we measured.

### S1.7 Experimental temporal resolution

In pump-probe ultrafast spectroscopy, the time resolution is commonly taken as the width of the instrumental response function (IRF), which is defined as the cross-correlation between the temporal intensity profiles of pump and probe pulses. Since the pump pulse duration (30 fs) is much faster than the probe pulse (1.30 ps = 1300 fs), this cross-correlation is dominated by the probe pulse duration, giving a full width half maximum (FWHM) for the IRF of 1300 fs fs, as illustrated in Figure S4a. This means that our temporal resolution is uniquely limited by the probe pulse. Other possible limiting factors, such jitter or temporal chirp are negligible in comparison to the probe pulse duration. On the one hand, according to the specifications of our laser system (SPIRIT-70 from Spectra Physics), jitter is negligible when the laser system is temperature-controlled, as in our case. On the other hand, temporal chirp, in which different frequency components travel at different speeds, resulting in a stretched pulse duration, may only affect the pump pulse, since this effect is negligible for pulse durations longer than 100 fs[11]. Our pump pulse is 30 fs and travels through a 25 mm BK7 cube beam splitter, elongating its pulse duration, and a few other optical elements. As seen in the Figure 2, for an 800 nm and a pulse duration of 30 fs, after traveling through 20 mm of BK7 material, the resulting pulse will still be shorter than 100 fs. Since our pump laser goes through a slightly longer distance of BK7 material, plus it travels for several meters through air and a few other 1mm optical elements, which can add a small contribution towards this temporal chirp, we simulated the cross-correlation function with a pump pulse of 500 fs, which is extremely conservative, and we still see that the IRF is dominated by the probe pulse duration of 1.30 ps, as seen in Figure S4b.

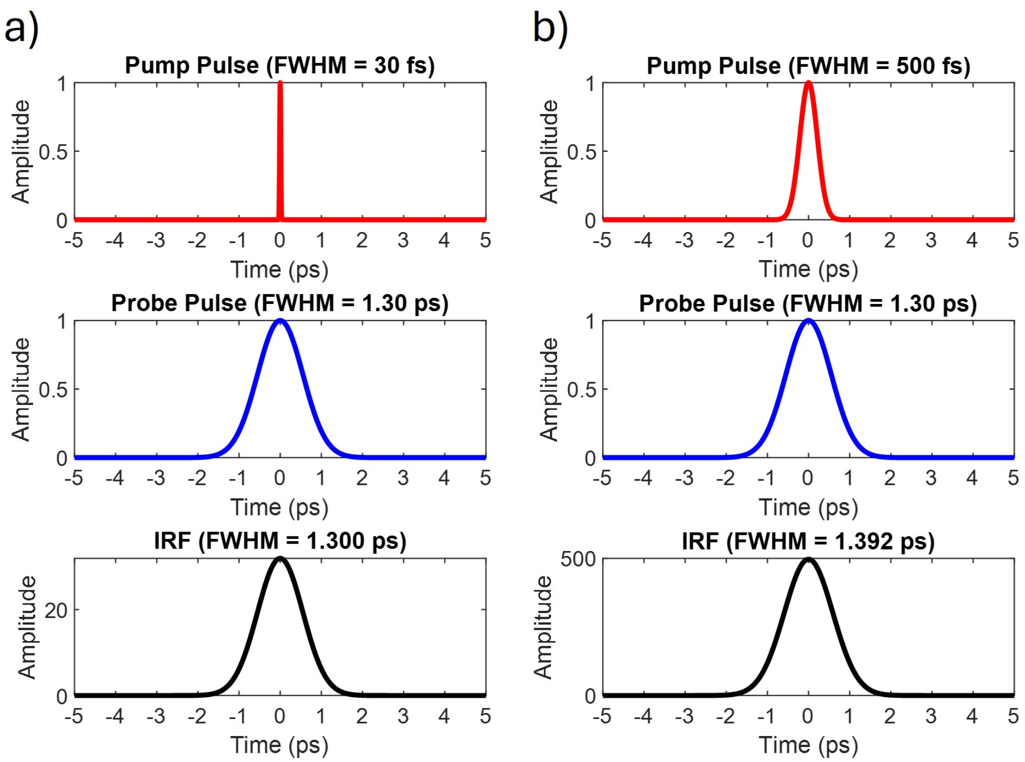


Figure S4: a) Instrument response function calculated from the cross-correlation between pump and probe pulses. Probe pulse is 1.30 ps and pump pulse is (a) 30 fs and (b) 500 fs.

## S1.8 Pre-pump excitation heating

Our experimental temperature calculations reveal a pre-heating before ultrafast excitation. This could have different origins that we exclude after performing relevant experiments:

- **Effect of leakage of pump light:** When performing both TRRS and TR, we filter the pump light with a spectral filter. However, a small portion of the pump light may still go through and affect our measurements. Yet, we have not detected any significant difference in the temperature at pre-time zero for measurements performed with different fluences (pump powers), indicating that there is no significant residual pump light affecting our results.

- **Effect of repetition rate:** If the repetition rate is faster than the time the system needs to return to equilibrium after pump excitation, there could be a contribution from pulse heat accumulation that would lead to an increased temperature before time zero. To test whether this affects our measurements, we performed a study for several repetition rates (1 MHz, 500 kHz, and 200 kHz). In the presence of heat accumulation, the Raman peak position of the germanium sample should shift toward lower frequencies as the repetition rate increases. However, we do not observe this, indicating no heating between pulses. Moreover, we see no deviations in the linewidth of the spectra as a function of repetition rate.

- **Sample degradation:** If the sample degraded after pump excitation, we would not obtain good reproducibility in our measurements. However, our measurements are long, so to ensure that no degradation or other artifacts such as laser alignment drift occur, we measure the time steps in a random fashion. That is, the time trace is not taken in chronological order but alternates between random time steps. In particular, the first points measured were in the following order: (-10 ps, -5 ps, 0 ps, 5 ps, 10 ps, -6.35 ps, -4.35 ps, -2.35 ps, -0.35 ps, 1.65 ps, etc.). This ensures that no unwanted artifacts affect our measurements. Figure S5 shows the Raman spectra at two different pre-excitation times separated by more than 3.5 hours. Both spectra overlap perfectly, indicating no sample degradation and good stability of the laser alignment.

Therefore, we can assign the observed 20K before pump excitation to probe heating. Its origin comes from the use of relatively large probe power, as compared to steady-state Raman spectroscopy, in which 100 times less power is enough to obtain a similar signal in comparable conditions. However, as the laser is pulsed with a repetition rate of 1 MHz, the actual time that the CCD camera is acquiring a signal corresponds to the duration of the probe pulse, about 1.30 ps. Then, the CCD camera continues to acquire data for a time corresponding to the time distance in between pulses, i.e., 1 $\mu$s. Therefore, we need to use relatively high probe power together with long exposure time to ensure that we measure a sufficient number of pulses, and take multiple accumulations. The fact that we are using relatively high probe power results in a constant probe-heating, that can be considered as a slightly higher base temperature. This heating is constant throughout the temporal window, and it does not affect the dynamics.

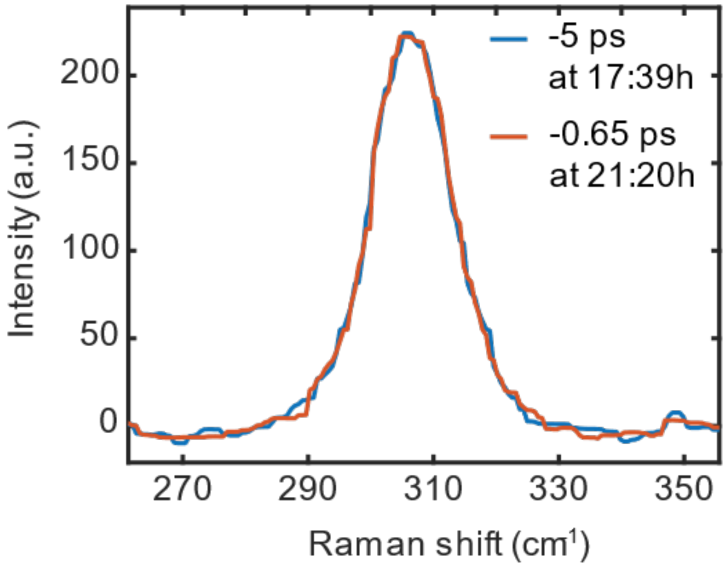


Figure S5: Raman spectra of germanium at two different time delays before pump excitation, taken with a time separation of more than 3.5 hours.

Furthermore, we show in the manuscript that higher pump fluence does not change the temperature dynamics and, therefore, it is reasonable that slight steady heating by the probe beam also does not affect the temperature dynamics but only the base temperature and, thereby, the initial phonon population.

## S2 Ab initio calculations of electron-phonon and phonon-phonon interactions

### S2.1 Computational details

In this work, germanium is described within density functional theory (DFT) and density functional perturbation theory (DFPT) using QUANTUM ESPRESSO, Wannier90, EPW and D3Q codes. Germanium was described within the LDA approximation, with a cutoff energy of 60 Ry and a 12x12x12 Γ-centered **k**-point grid. The lattice parameter of 10.696 a.u.[12] was used in all the calculations with the QUANTUM ESPRESSO package[13].

The Wannierization parameters used for the Wannier90 code[14] were as follows: 8 Wannier functions were constructed from 10 initial Bloch states, using atom-centered $sp^3$ projections as the initial guess. A disentanglement window of 17 eV and a frozen window of 12.203 eV were applied, along with a 16 × 16 × 16 Γ-centered **k**-point grid. Disentanglement convergence was achieved within 109 iterations, with a tolerance of $10^{-12}$ Å$^2$, while the spread minimization reached a tolerance of $10^{-10}$ Å$^2$ after 200 iterations. The total spread for all eight Wannier functions was 33.45 Å$^2$. For the interpolation of the electron-phonon matrix elements within the EPW code[15], we used the above parameters and a 8x8x8 **q**-point grid.

For the calculation of LO/TO phonon decay via phonon-phonon interaction, we used D3Q code[16], un-

der a very fine internal grid of 39 × 39 × 39 q-points and a Gaussian smearing of 2 cm$^{-1}$.

## S2.2 Energy transfer from carriers to phonons

The rate of energy transfer from a single carrier to phonons reads[17]:

$$\begin{aligned} \frac{\delta E}{\delta t}_{n\mathbf{k}} = \\ 2\pi \sum_{m\nu} \int \frac{d\mathbf{q}}{\Omega_{BZ}} \omega_{\mathbf{q}\nu} |g_{mn\nu}(\mathbf{k},\mathbf{q})|^2 (1 - f_{m,\mathbf{k+q}})(N_{\mathbf{q},\nu}+1)\delta(\varepsilon_{n,\mathbf{k}} - \varepsilon_{m,\mathbf{k+q}} - \hbar\omega_{\mathbf{q}\nu}) - \\ -2\pi \sum_{m\nu} \int \frac{d\mathbf{q}}{\Omega_{BZ}} \omega_{\mathbf{q}\nu} |g_{mn\nu}(\mathbf{k},\mathbf{q})|^2 (1 - f_{m,\mathbf{k+q}}) N_{\mathbf{q},\nu} \delta(\varepsilon_{n,\mathbf{k}} - \varepsilon_{m,\mathbf{k+q}} + \hbar\omega_{\mathbf{q}\nu}) \end{aligned} \quad (8)$$

Here, $g_{mn\nu}(\mathbf{k},\mathbf{q})$ is the electron-phonon matrix element, which depends on the initial electronic state $|n,\mathbf{k}\rangle$ with band number $n$ and wavevector $\mathbf{k}$, on the phonon $|\nu,\mathbf{q}\rangle$, where $\nu$ is phonon mode number and $\mathbf{q}$ phonon wave vector, and on the final electronic state $|m,\mathbf{k+q}\rangle$. The Dirac delta functions $\delta(\varepsilon_{n,\mathbf{k}} - \varepsilon_{m,\mathbf{k+q}} - \hbar\omega_{\mathbf{q}\nu})$ and $\delta(\varepsilon_{n,\mathbf{k}} - \varepsilon_{m,\mathbf{k+q}} + \hbar\omega_{\mathbf{q}\nu})$ represent the energy conservation laws for respectively phonon emission and absorption. $f_{n,\mathbf{k}}$ is the carrier distribution function. Note that the phonon occupations $N_{\mathbf{q},\nu}$ depend on lattice temperature $T_L$, while the carrier Fermi-Dirac distribution function depends on carrier temperature $T_c$ and on the chemical potential $\mu$.

The total rate of energy transfer from carriers to phonons for a given initial carrier distribution reads:

$$\frac{\delta E}{\delta t}_{c-ph} = \sum_{n} \int \frac{d\mathbf{k}}{\Omega_{BZ}} \frac{\delta E}{\delta t}_{n\mathbf{k}} f_{n,\mathbf{k}} \quad (9)$$

The rate of energy transfer $\frac{\delta E}{\delta t}_{n\mathbf{k}}$ from photoexcited holes close to valence band maximum to TO/LO phonons was calculated using modified EPW code on 30x30x30 $\mathbf{q}$-grid (convergence checked with 50x50x50 grid). An example of such calculation is shown in Figure S6, where calculated $\frac{\delta E}{\delta t}_{n\mathbf{k}}$ for $\mathbf{k}$-points belonging to 100x100x100 grid is shown as a function of hole excess energy $\epsilon$ for hole distribution with $T_c = 2500$ K and $\mu = -0.03$ eV, which is close to the valence band maximum (shown in green dots). The rate of energy transfer from carriers to phonons changes with excess energy as the density of final states (DOS), as already reported in previous works (see e.g.[18]). Because of this, the calculated *ab initio* data can be successfully modeled with energy-dependent model of the form $\frac{\delta E}{\delta t}(\epsilon) = C_{el-ph}(1 - f(\epsilon))\sqrt{\epsilon}$, where $C_{el-ph}$ is a constant. This can be seen in Figure S6: the model (clear green line) and the *ab initio* data are hardly distinguishable for excess energies below 1 eV. Note that the excess energies of interest in this study do not exceed 0.5 eV. The Pauli blocking factor $1 - f(\epsilon)$ plays an important role at high temperatures and low excess energies, whereas for $T_c = 300$ K (black dashed line), the square-root dependence of the rate of energy transfer on the excess energy is clearly visible. Note that once the $C_{el-ph}$ is obtained from DFT calculation and validated by comparing DFT and model results, the hot hole dynamics can be described in the framework of the two-temperature excess-energy dependent model as described below.

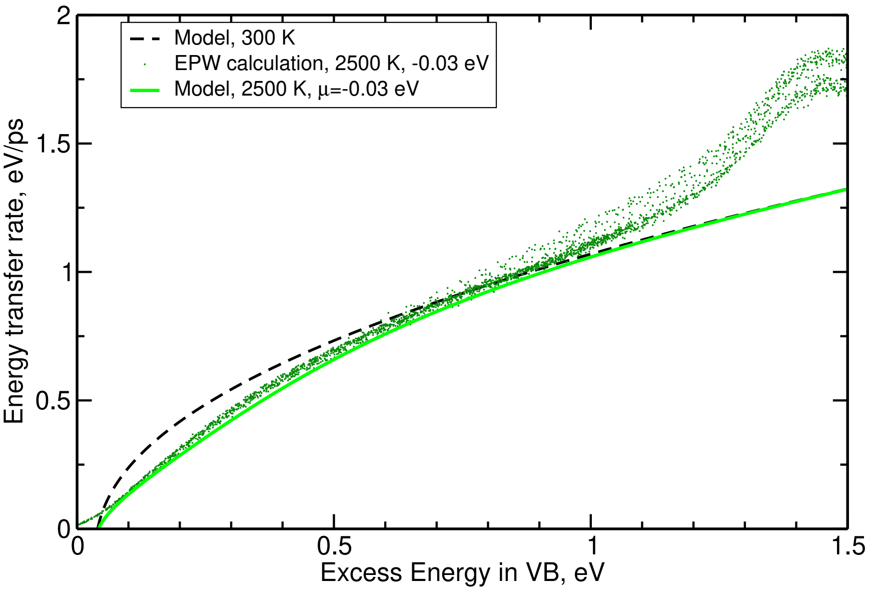


Figure S6: The rate of energy transfer $\frac{\delta E}{\delta t}_{n\mathbf{k}}$ from photoexcited holes close to valence band maximum to TO/LO phonons calculated using modified EPW code, and compared to the excess-energy dependent model $\frac{\delta E}{\delta t}(\epsilon)$ (see text).

## S2.3 Hot hole dynamics

The hot hole dynamics is described by propagating in time the coupled time-dependent Boltzmann transport equations (t-BTE) for carriers and phonons, in the framework of excess-energy dependent model which was described in[18]. In the case of the thermalised distributions which we consider in this work, this model is equivalent to two-temperature model[19]. The detailed expressions for carrier-phonon collision term can be found e.g. in[18].

The t-BTE for carriers reads:

$$\frac{\delta f(\epsilon)}{\delta t} = \left.\frac{\partial f(t)}{\partial t}\right|_{\mathrm{el-c}} \tag{10}$$

Here, $t$ is time. The collision term on the right-hand side accounts for carrier-phonon interactions.

The t-BTE for phonons reads:

$$\frac{\delta N_{TO/LO}(T_L)}{\delta t} = \left.\frac{\partial N_{TO/LO}(T_L,t)}{\partial t}\right|_{\mathrm{coll}} \tag{11}$$

Here, the phonons are represented by one effective TO/LO phonon mode. The collision term on the right-hand side accounts for phonon-hole interactions which lead to the increase of phonon temperature and for the decay of TO/LO modes into acoustic phonons, with the decay constant calculated *ab initio* with the D3Q code. The coupled t-BTEs for carriers (holes) and phonons are solved by time-stepping with a time step of 1 fs. Note that, as the holes transfer energy only to the part of the Brillouin zone (BZ) close to the zone center, the Raman-active optical phonons in this part of the BZ become overheated with respect to average temperature of optical phonons in the BZ. To account for this, we define the "local" temperature of the Raman-active modes $T_R$: $T_R = \frac{\Omega_{BZ}}{\Omega_R} T_L$. Here, $\frac{\Omega_{BZ}}{\Omega_R}$ is the ratio between BZ volume and the heated part of BZ. This coefficient is determined from the calculated carrier-phonon **q**-dependent spectral function for energy transfer (per carrier), which is defined as follows:

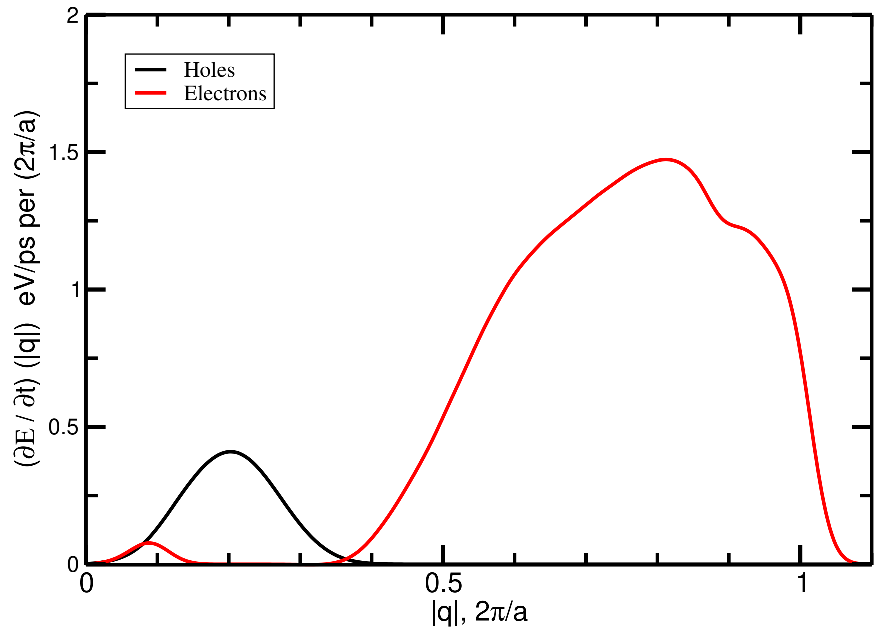


Figure S7: The **q**-dependent spectral functions for the energy transfer between carriers and optical phonons (see text), calculated per carrier for electrons and for holes in Ge.

$$\frac{\delta E}{\delta t}(|\mathbf{q}|) = 2\pi \sum_{m\nu} \int\int \frac{d\mathbf{q}'}{\Omega_{BZ}} \frac{\delta E}{\delta t}_{\mathbf{k},\mathbf{k}+\mathbf{q}'} \delta(|\mathbf{q}| - |\mathbf{q}'|) \tag{12}$$

Examples of calculated **q**-dependent spectral function for the energy transfer between carriers and optical phonons is shown in Figure S7 for holes and for electrons in Ge. In our calculations, $\frac{\Omega_{BZ}}{\Omega_R}$ for holes is considered to be between 3.5 and 4, which corresponds to the heating of TO/LO phonons in 25-30% of BZ, as one can see in Figure S7. Figure S7 also shows why the heating of Raman-active modes is dominated by holes: as one can see, electrons transfer their energy to large-**q** (intervalley) phonons.

# S3 Molecular Dynamics calculations

## S3.1 Simulation setup

## S3.2 Generalized Langevin Equation excitation

Atoms in the central region were excited by means of the GLE thermostat[20;21;22]. The GLE implements non-Markovian dynamics by introducing history-dependent terms[23] in the standard Langevin equation. In this implementation, it is designed with a standard white noise Langevin thermostat with friction $\gamma_{\text{base}}$ and target temperature $T_{\text{base}}$, and a $\delta-$thermostat[24] at a temperature $T_{\text{exc}}$ that is coupled with a friction parameter $\gamma_{\text{exc}}$. The memory kernel power spectrum reads as

$$K(\omega) = 2\gamma_{\text{base}} + \frac{\gamma_{\text{exc}}}{\pi} \frac{\omega_{\text{exc}} \Delta\omega\omega^2}{(\omega^2 - \omega_{\text{exc}}^2)^2 + \Delta\omega^2\omega^2} \tag{13}$$

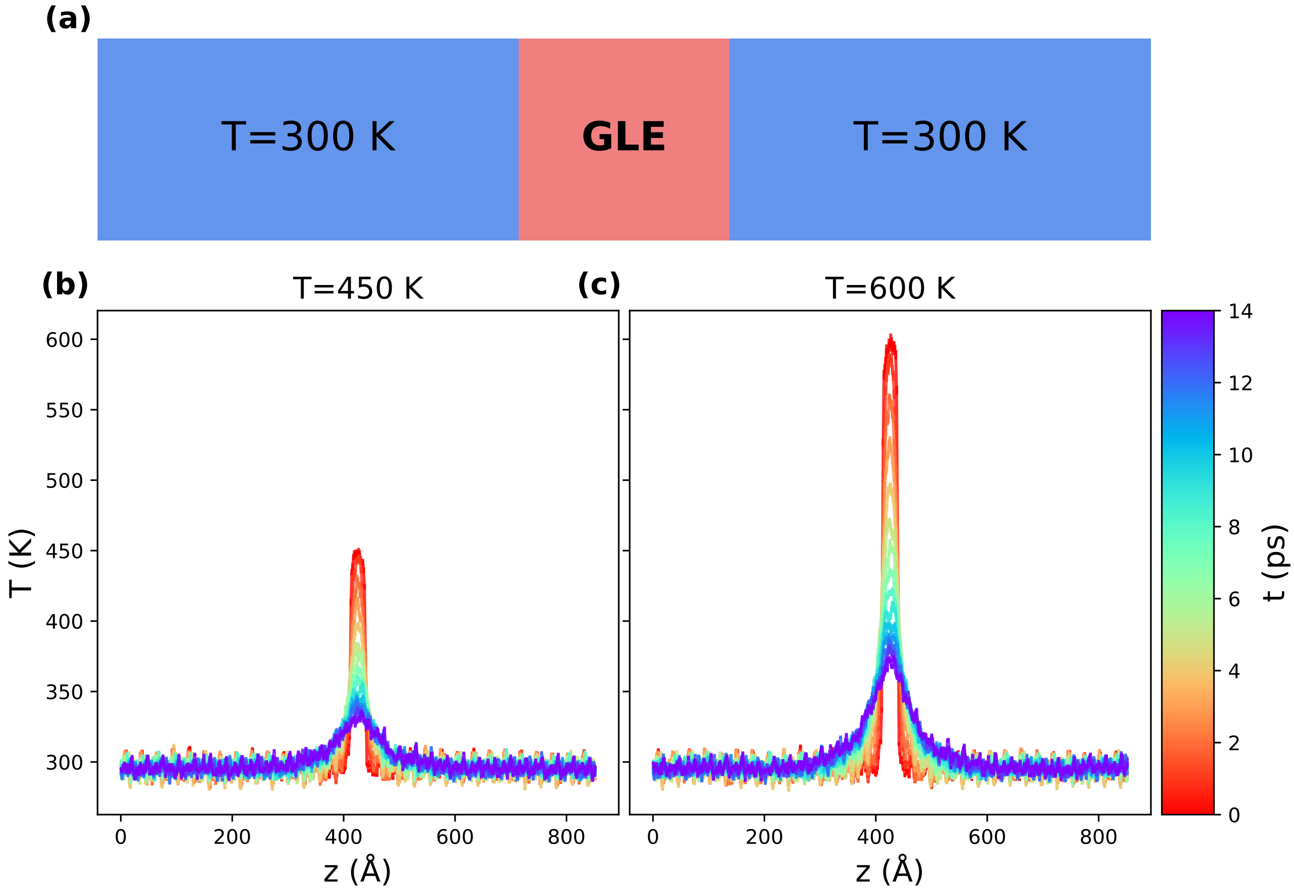


Figure S8: a) Schematic representation of the simulation cell used in the MD simulations. b-c) Temperature profile as a function of time during the relaxation for the two excitation temperature considered in our simulations.

The friction parameters were chosen to be $\gamma_{\text{exc}} = 0.5$ ps$^{-1}$ and $\gamma_{\text{base}} = 0.5$ ps$^{-1}$. The thermostat was tuned at the optical phonon peak frequency for the Tersoff potential, $\omega_{\text{exc}} = 310$ cm$^{-1}$, while the frequency window was chosen as $\Delta\omega = 0.1$ cm$^{-1}$ in order to minimize any energy spill-over arising from unavoidable mode coupling, which does not guarantee a perfect adiabatic thermalization of the stretching mode. However, such a coupling does not interfere with the relaxation dynamics[22].

The system excitation and the relaxation is mainly monitored using the kinetic temperature, while the spectral features were extracted from a time-resolved vibrational density of states (vDOS) that is calculated every 500 fs by means of a short-time Fourier transform of the atomic velocity autocorrelation function (VACF) as

$$g(\omega, t) = \frac{1}{2\pi\, N} \sum_{i=1}^{N} \int_{t_1}^{t_2} w(\tau) \left\langle \mathbf{v}_i(t) \cdot \mathbf{v}_i(t+\tau) \right\rangle e^{-i\omega\tau}\, d\tau \tag{14}$$

where the VACF is evaluated at the time $t$, $w(\tau)$ is a real–valued window function (Blackman-Nuttall[25]) of width $\Delta t = t_2 - t_1$ that localizes the Fourier transform around $t$, and $N$ is the total number of atoms. The $g(\omega, t)$ is then used to calculate the intensity of the peaks and the frequency shifts as a function of

time as

$$\mathcal{I}(\omega,t) = \int_{\omega_1}^{\omega_2} g(\omega',t)d\omega'$$
$$\omega(t) = \frac{\int_{\omega_1}^{\omega_2} \omega' g(\omega',t)d\omega'}{\int_{\omega_1}^{\omega_2} g(\omega',t)d\omega'} \tag{15}$$

## S3.3 NVE relaxation

**Kinetic temperature** During the relaxation we can monitor the system evolution by computing the kinetic temperature difference between the two regions, as shown in Figure S9.

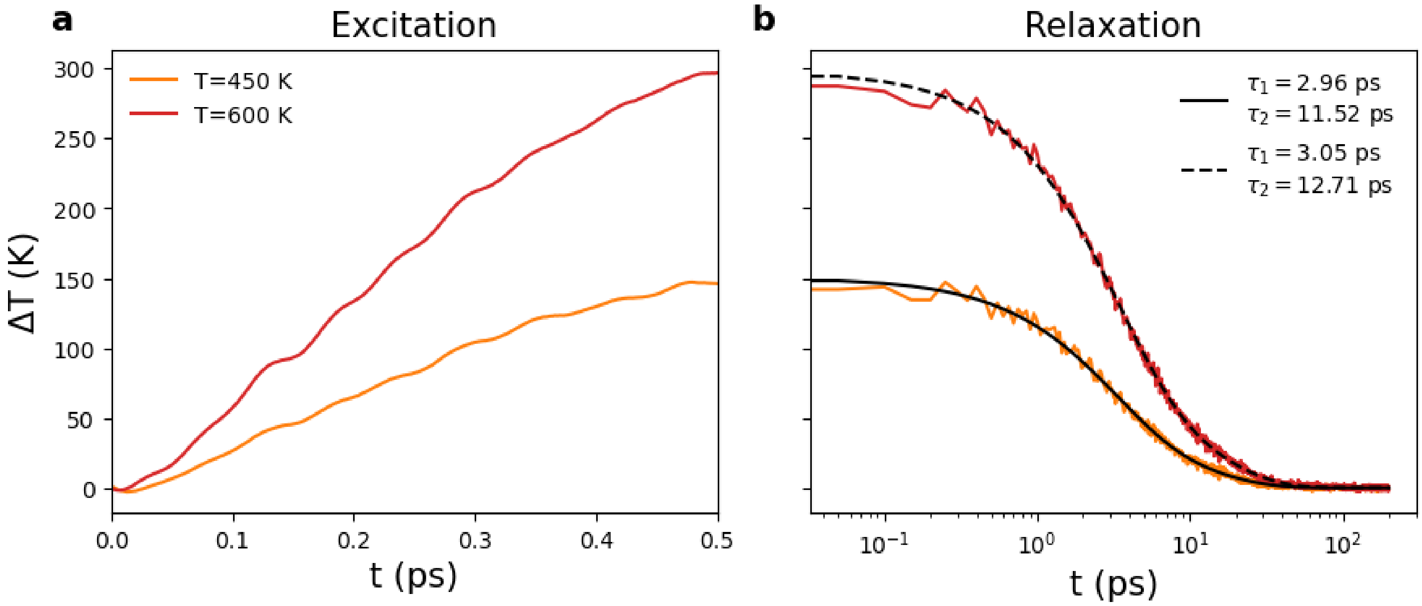


Figure S9: Temperature difference between the excited and unexcited regions during (a) the 500 fs-long excitation, and (b) during the relaxation. Here temperatures are reported with a log scale along with their fit (dashed black lines) using a two exponential model.

Panel a shows the temperature rise during the 500 fs excitation for both cases, efficiently reaching $T_{\text{exc}} =$ 450 K and 600 K. In panel b we report the temperature difference during the relaxation phase: as anticipated in the main text, it follows a two-times exponential decay which we fit with the black-dashed curve. In particular, the fast decay occurs within $\sim$ 3 ps reflecting the phonon thermalization dynamics. The second slower process suggest instead a spatial thermal diffusion from the locally heat area to the unperturbed regions.

**Spectral features** As mentioned in the main text, the GLE inject energy in a specific frequency interval resulting in an enhanced intensity of the corresponding peak in the vDOS. During the NVE, this excess energy will be quickly redistributed within the vibrational modes of the excited region atoms, and this process will dominate the initial phase of the relaxation. A representation of this phenomenon is reported in Figure S10.

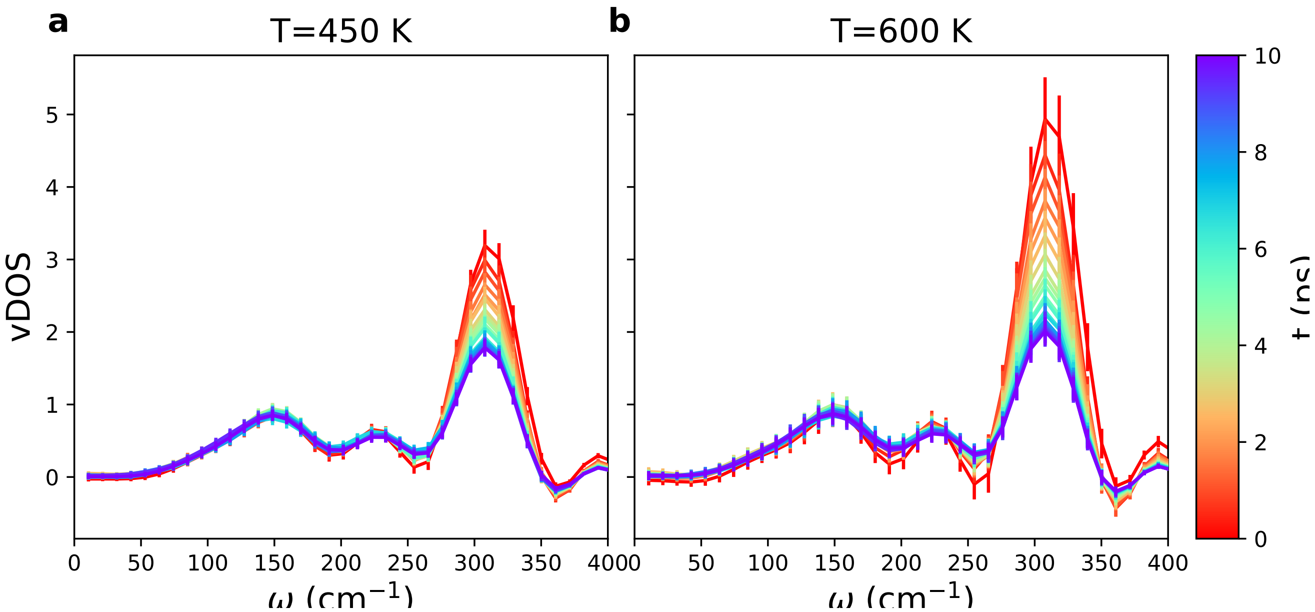


Figure S10: Vibrational density of states during the relaxation as a function of time for (a) $T_{\mathrm{exc}} = 450$ K, and (b) 600 K

Consistent with the experimental analysis, we performed the extraction of the spectral features for the optical peak by considering the frequency interval between 260 and 340 cm$^{-1}$. In Figure S11a and b we show the extracted intensity of the excited optical peak as a function of time, using the intensity equation in Equation (15), and fitted using a two-times exponential decay.

Frequency shift was extracted by performing a weighted average as suggested in Equation (15). As commented in the main text, the frequency decays following a single exponential and with a time scale that agrees with the spatial thermal diffusion identified in Figure S9. Furthermore, we found that the frequency shift is convoluted with oscillations, as suggested by the presence of regularly spaced peaks in Figure S11c and d. These are closely related to the thermal expansion caused by the local heat of the excitation, and will be commented more in detail when illustrating the atomic stress calculations. Finally, as mentioned in the main text, Figure S11e and f show the FWHM of the excited peak as a function of time: the time evolution follows a single exponential decay, with a somehow intermediate timescale with respect to the peak intensity and the peak frequency: it results from an interplay between the strong anhamornicity induced by the excitation and the heating effect following the system relaxation. To illustrate the phonon thermalization via mode coupling, we calculated the peak intensity for the mid-range frequency peak at $\sim$ 220 cm$^{-1}$ and for the acoustic region of the vibrational spectrum centered around 150 cm$^{-1}$. As explained in the main text, these intensities decay with a two-times exponential decay but with a fast rise (within few ps) and a following slow decay (Figure S12 and Figure S13). We infer that the fast rise is the fingerprint of phonon scattering originating from optical modes, while the slower component is again a reflection of the thermal diffusion following the initial relaxation.

These data are significantly noisier than the ones obtained from the optical peak: the peaks are broader and less intense, thus much more sensitive to the numerical uncertainty. In fact, the optical phonons will

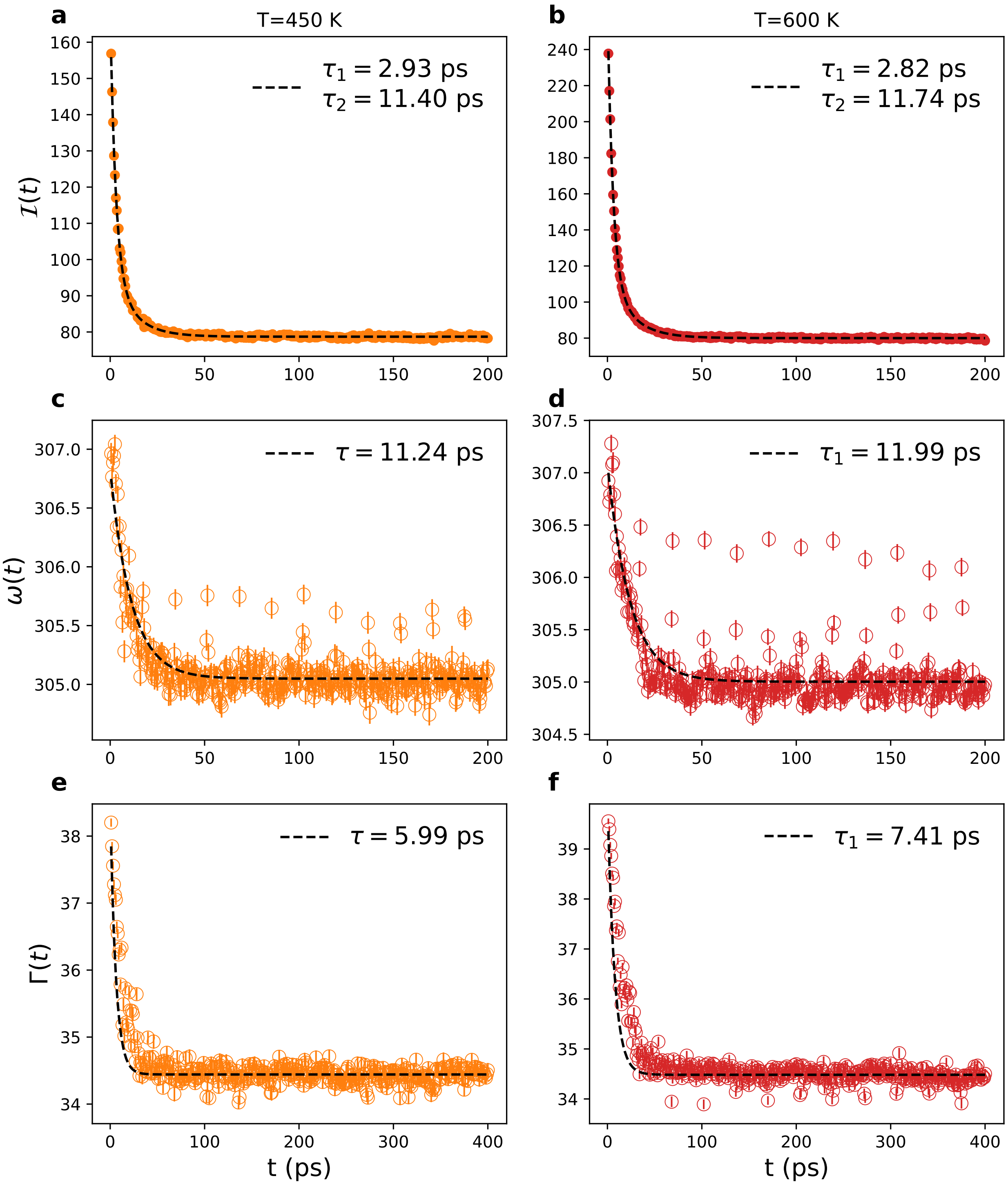


Figure S11: Time dependence of the extracted spectral features for $T_{\mathrm{exc}} = 450$ K (left column), and 600 K (right column). a-b) Excited peak intensity along with the two exponential model fit; c-d) Excited peak frequency along with the single exponential model fit; e-f) Excited peak FWHM along with the single exponential model fit.

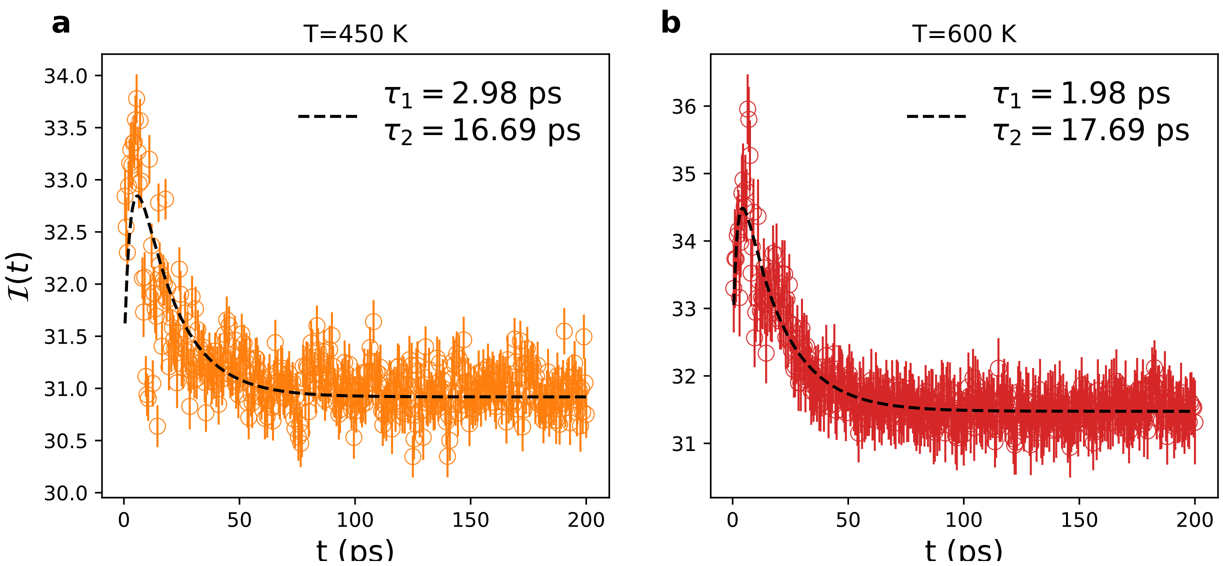


Figure S12: Time dependence of the 220 $cm^{-1}$ peak intensity along with the two exponential model fit for (a) $T_{exc}$ = 450 K, and (b) 600 K.

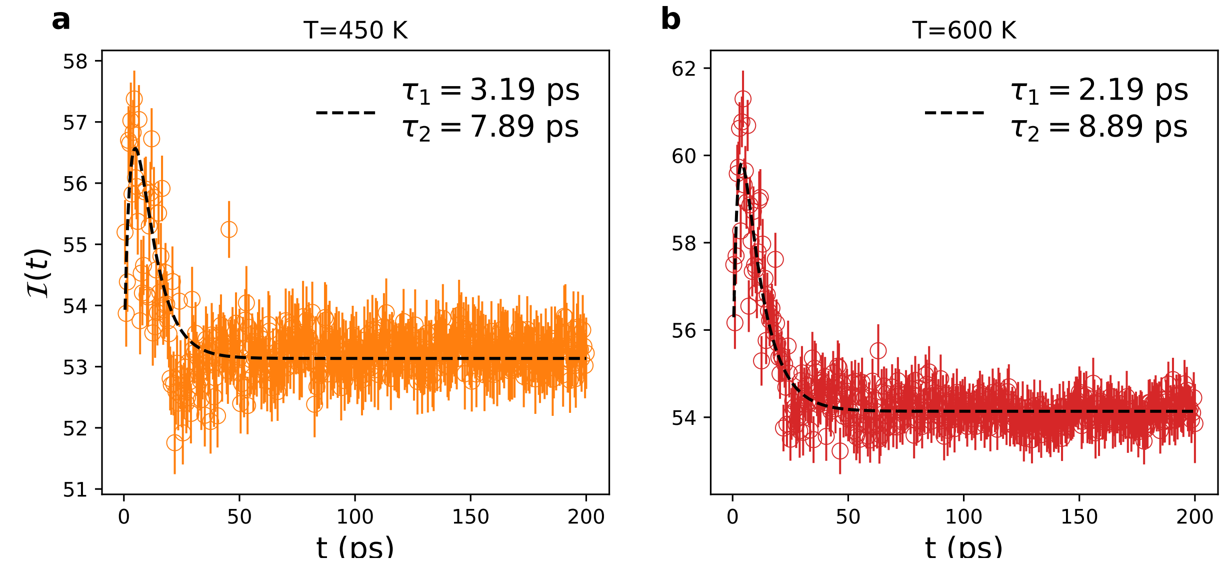


Figure S13: Time dependence of the 150 $cm^{-1}$ peak intensity along with the two exponential model fit for (a) $T_{exc}$ = 450 K, and (b) 600 K.

scatter with the lower frequency phonons of the atoms in the excited spot but also with the surrounding atoms from the unexcited regions. For this reason, the excess energy is transferred to a larger reservoir of atoms than the one we are using to extract the spectral features (that we remark being extracted by considering only the excited atoms). In other words, to obtain the peak intensity for the lower frequency modes there is less available vibrational energy per-atom.

**Atomic stress** As mentioned in the main text, an important role in the relaxation of the system is played by the thermal expansion caused by the local heat following the excitation. To quantify this aspect, we

used the per-atom stress tensor as computed by LAMMPS. The tensor is computed using the virial theorem, thus accounting for both kinetic and potential contributions

$$S_i^{\alpha\beta} = -\left(m_i v_i^\alpha v_i^\beta + \sum_{j \neq i} r_{ij}^\alpha F_{ij}^\beta\right) \tag{16}$$

where $\alpha, \beta \in \{x, y, z\}$ are Cartesian components, $m_i$ is the mass of atom $i$, $v_i^\alpha$ is the $\alpha-$component of the velocity of atom $i$, $r_{ij}^\alpha$ is the $\alpha-$component of the displacement vector from atom $i$ to atom $j$, and $F_{ij}^\beta$ is $\beta-$component of the force exerted on atom $i$ by atom $j$. By convention, a positive stress means tension and a negative stress means compression. The atomic stress is averaged over all atoms in the two sub-regions to obtain an atomic stress difference as a function of time.

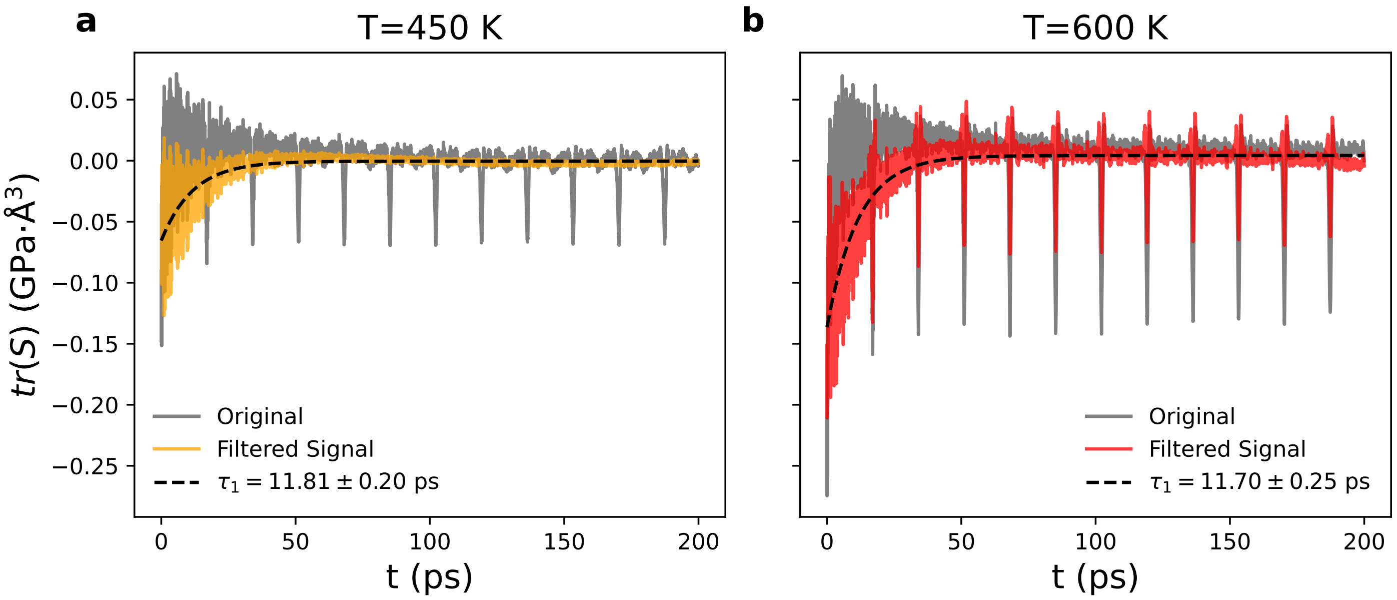


Figure S14: Time dependence of the atomic stress difference between the excited and unexcited region (grey curve), the filtered data using the Savitzky-Golay filter (orange and red curves) along with the single exponential model fit for (a) $T_{\text{exc}} = 450$ K, and (b) 600 K.

Figure S14 shows the on-the-fly computed atomic stress during the NVE run (grey curves): it starts from a negative value (the excited region tends to expand due to the higher temperature) approaching zero as the equilibrium is reached. However, the raw data present some regular spikes which are the consequence of size effects: as a consequence of an initial thermal gradient and stress imbalance in the simulation cell, collective vibrational modes of atoms propagate mechanical stress through the lattice. Due to the finiteness of the simulation cell, they reflect off periodic images, and interfere constructively or destructively with ongoing stress oscillations. Since these spikes occurs approximately every 15 ps, using an average speed of sound for the Tersoff potential ($\sim$ 3800 m/s) we get $v_s T/2 \approx 2.8$ nm which is the transverse section of our sample, confirming the relation between these oscillations and the finiteness the simulation cell. These were filtered out using the Savitzky-Golay filter (although for $T = 600$ K they could not be completely eliminated) and we fitted the resulting stress relaxation with a single exponential: the relax-

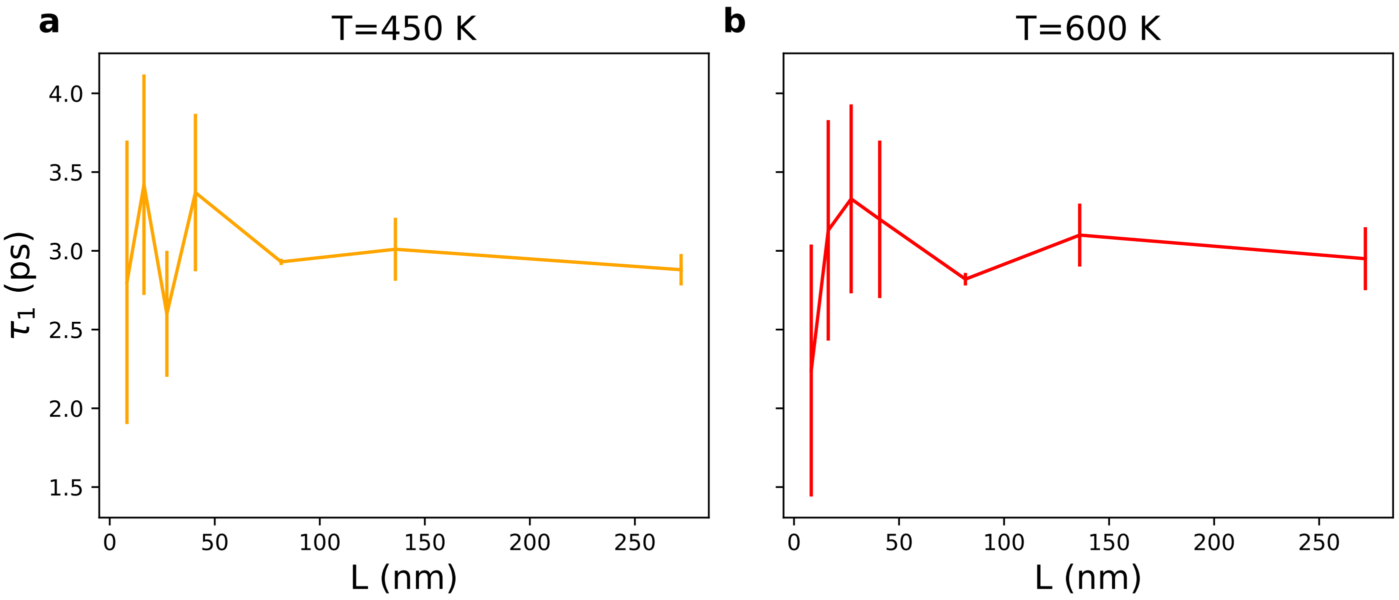


Figure S15: $\tau_1$ calculated from the excited peak intensity decay as a function of system size for (a) $T_{\mathrm{exc}} = 450$ K, and (b) 600 K.

ation time of $11.8 \pm 0.2$ ps ($11.7 \pm 0.3$) for $T = 450$ K ($T = 600$ K), correspond to the time-scale observed in the frequency shift, hence confirming the role played by thermal expansion.

### S3.4 Size effects

As mentioned in the previous section, non-equilibrium MD simulation are often affected by size-effect due to the unavoidable finiteness of simulation boxes. Depending on the observable different approaches can be adopted to address this issue, but it is fundamental to perform convergence tests. For this reason, we calculated the decay time of the excited optical peak as a function of the simulation cell length: we varied the the length keeping the width of the excited spot unchanged, basically varying the size ratio between the excited and unexcited region. In fact, in a laboratory setup the laser excites a region which is infinitely smaller than the sample size, which acts a infinite ”reservoir”. To understand what length scale allows a proper investigation of the relaxation dynamics, we report in Figure S15 the fast decay time (identifying the phonon thermalization process) as a function of the cell length. In fact, as previously observed[25], vibrational relaxation and thermal diffusion typically occur on different scales and the two process cannot be efficiently decoupled if not considering the proper distances.
From our tests we conclude that the $\tau_1$ decay time doesn't vary appreciably for $L > 50$ nm, hence we considered simulation cells 82.1 nm-long as a compromise between accuracy and computational workload.

## S3.5 Temperature effects

The observed behavior during the system relaxation is expected to hold over a wide range of temperatures. We performed additional benchmarks at different excitation temperatures, obtaining similar trends for the kinetic temperature and the optical peak, as shown in Figure S16 and Figure S17, respectively.

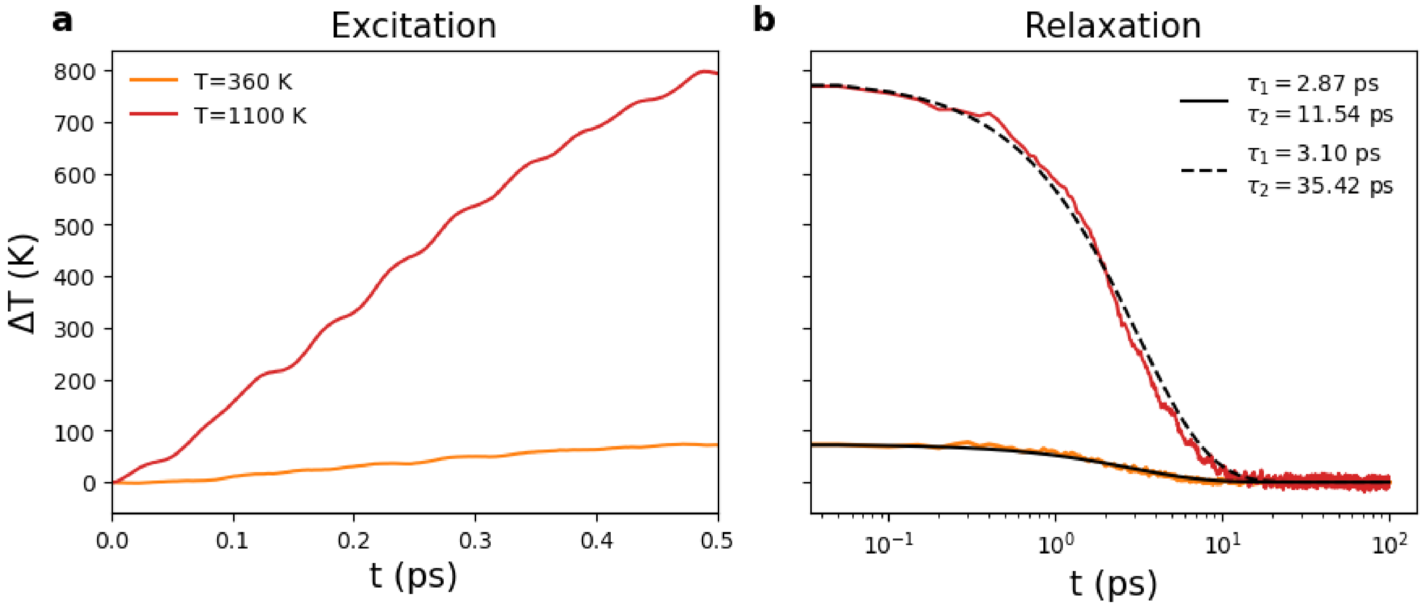


Figure S16: Temperature difference between the excited and unexcited regions during (a) the 500 fs-long excitation, and (b) during the relaxation, for $T_{exc} = 360$ K and $T_{exc} = 1100$ K.

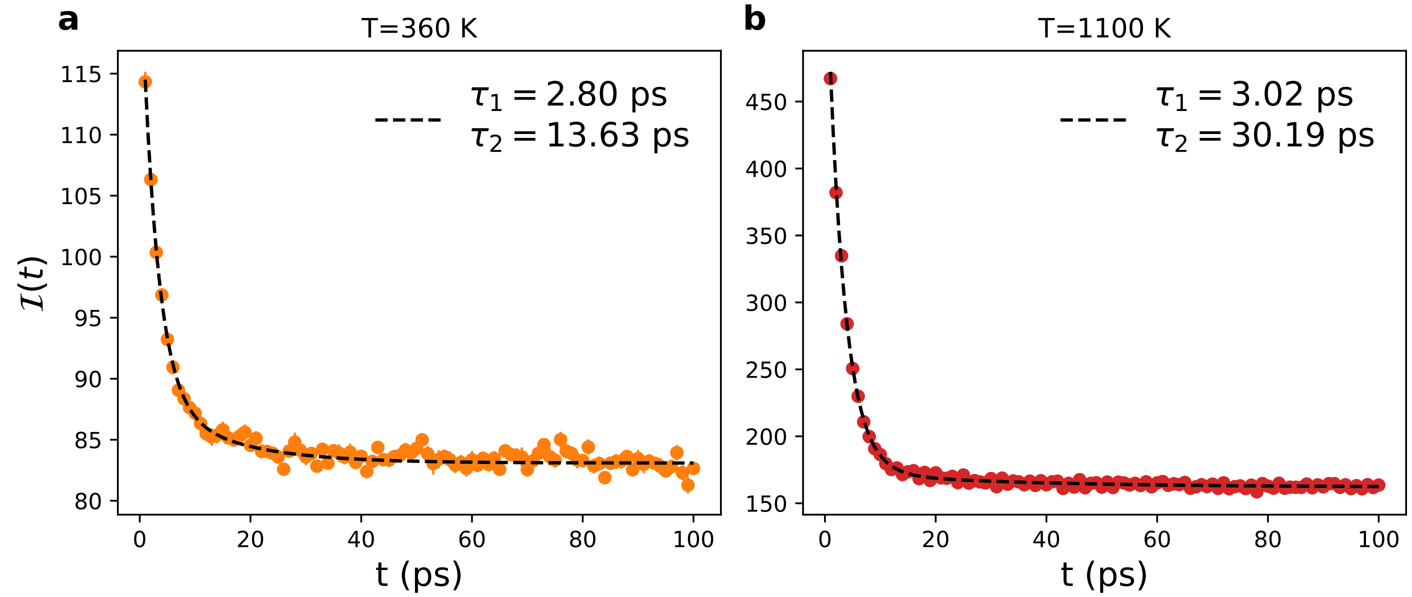


Figure S17: Time dependence of the excited peak intensity for (a) $T_{exc} = 360$ K and (b) $T_{exc} = 1100$ K.

# References


[1] L. Tian, L. Di Mario, A. K. Sivan, D. Catone, P. O'Keeffe, A. Paladini, S. Turchini, F. Martelli, *Nanotechnology* **2019**, *30*, 21 214001.

[2] P. Hauer, J. Grand, A. Djorovic, G. R. Willmott, E. C. Le Ru, *The Journal of Physical Chemistry C* **2016**, *120*, 37 21104.

[3] J. Menéndez, M. Cardona, *Phys. Rev. B* **1984**, *29* 2051.

[4] H. Tang, I. P. Herman, *Phys. Rev. B* **1991**, *43* 2299.

[5] R. P. Prasankumar, A. J. Taylor, editors, *Optical Techniques for Solid-State Materials Characterization*, CRC Press, Boca Raton, **2012**.

[6] S. Sandell, E. Chávez-Ángel, A. El Sachat, J. He, C. M. Sotomayor Torres, J. Maire, *Journal of Applied Physics* **2020**, *128*, 13 131101.

[7] K. S. Olsson, K. An, X. Li, *Journal of Physics D: Applied Physics* **2018**, *51*, 13 133001.

[8] Y. Ezzahri, S. Grauby, J. M. Rampnoux, H. Michel, G. Pernot, W. Claeys, S. Dilhaire, C. Rossignol, G. Zeng, A. Shakouri, *Phys. Rev. B* **2007**, *75* 195309.

[9] O. B. Wright, *Journal of Applied Physics* **1992**, *71*, 4 1617.

[10] D. E. Aspnes, A. A. Studna, *Physical Review B* **1983**, *27*, 2 985, publisher: American Physical Society.

[11] D. Polli, D. Brida, S. Mukamel, G. Lanzani, G. Cerullo, *Phys. Rev. A* **2010**, *82* 053809.

[12] V. Tyuterev, S. Obukhov, N. Vast, J. Sjakste, *Phys. Rev. B* **2011**, *84* 035201.

[13] P. Giannozzi, O. Andreussi, T. Brumme, O. Bunau, M. Buongiorno Nardelli, M. Calandra, R. Car, C. Cavazzoni, D. Ceresoli, M. Cococcioni, N. Colonna, I. Carnimeo, A. Dal Corso, S. de Gironcoli, P. Delugas, R. A. DiStasio, A. Ferretti, A. Floris, G. Fratesi, G. Fugallo, R. Gebauer, U. Gerstmann, F. Giustino, T. Gorni, J. Jia, M. Kawamura, H.-Y. Ko, A. Kokalj, E. Küçükbenli, M. Lazzeri, M. Marsili, N. Marzari, F. Mauri, N. L. Nguyen, H.-V. Nguyen, A. Otero-de-la Roza, L. Paulatto, S. Poncé, D. Rocca, R. Sabatini, B. Santra, M. Schlipf, A. P. Seitsonen, A. Smogunov, I. Timrov, T. Thonhauser, P. Umari, N. Vast, X. Wu, S. Baroni, *Journal of Physics: Condensed Matter* **2017**, *29*, 46 465901.

[14] G. Pizzi, V. Vitale, R. Arita, S. Blugel, F. Freimuth, G. Géranton, M. Gibertini, D. Gresch, C. Johnson, T. Koretsune, J. Ibañez-Azpiroz, H. Lee, J. M. Lihm, D. Marchand, A. Marrazzo, Y. Mokrousov, J. I. Mustafa, Y. Nohara, Y. Nomura, L. Paulatto, S. Poncé, T. Ponweiser, J. Qiao, F. Thole, S. S. Tsirkin, M. Wierzbowska, N. Marzari, D. Vanderbilt, I. Souza, A. A. Mostofi, J. R. Yates, *J. Phys.: Condens. Matter* **2020**, *32* 165902.

[15] S. Ponce, E. R. Margine, C. Verdi, F. Giustino, *Comp. Phys. Comm.* **2016**, *209* 116.

[16] L. Paulatto, F. Mauri, M. Lazzeri, *Phys. Rev. B* **2013**, *87* 214303.

[17] P. Allen, *Phys. Rev. Lett.* **1987**, *59* 1460.

[18] J. Sjakste, R. Sen, N. Vast, J. Saint-Martin, M. Ghanem, P. Dollfus, F. Murphy-Armando, J. Kanasaki, *The Journal of Chemical Physics* **2025**, *162*, 6 061002.

[19] F. Caruso, D. Novko, *ADVANCES IN PHYSICS: X* **2022**, *7* 2095925.

[20] M. Ceriotti, M. Parrinello, *Procedia Computer Science* **2010**, *1*, 1 1607, iCCS 2010.

[21] M. Ceriotti, G. Bussi, M. Parrinello, *Phys. Rev. Lett.* **2009**, *102* 020601.

[22] R. Dettori, M. Ceriotti, J. Hunger, C. Melis, L. Colombo, D. Donadio, *Journal of Chemical Theory and Computation* **2017**, *13*, 3 1284.

[23] R. W. Zwanzig, *Nonequilibrium Statistical Mechanics*, Oxford University Press, New York, NY, **2001**.

[24] M. Ceriotti, M. Parrinello, *Procedia Computer Science* **2010**, *1*, 1 1607–1614.

[25] R. Dettori, M. Ceriotti, J. Hunger, L. Colombo, D. Donadio, *The Journal of Physical Chemistry Letters* **2019**, *10*, 12 3447.
